# Determining the Impact of a Physiotherapist-Led Primary Care Model for Low Back Pain: Protocol and Analysis Plan for a Cluster Randomized Controlled Trial and Embedded Process Evaluation

**DOI:** 10.2196/89004

**Published:** 2026-02-26

**Authors:** Jordan Miller, Catherine Donnelly, Chad McClintock, Kevin Varette, Yeimi Camargo, Jacquelyn Marsh, Monica Taljaard, Mir Sanim Al Mamun, Geneviève Bacchus, David Barber, Lynn Cooper, Simon French, Jonathan Hill, Michael Green, Joy MacDermid, Kathleen Norman, Julie Richardson, Joan Tranmer, Timothy Wideman

**Affiliations:** 1 School of Rehabilitation Therapy Faculty of Health Sciences Queen's University Kingston, ON Canada; 2 Health Services and Policy Research Institute Faculty of Health Sciences Queen's University Kingston, ON Canada; 3 School of Physical Therapy Western University London, ON Canada; 4 Ottawa Hospital Research Institute Ottawa, ON Canada; 5 Epidemiology and Public Health University of Ottawa Ottawa, ON Canada; 6 Department of Family Medicine Faculty of Health Sciences Queen's University Kingston, ON Canada; 7 Canadian Injured Worker's Alliance Thunder Bay, ON Canada; 8 Spinal Pain Research Centre Department of Chiropractic Macquarie University Sydney, New South Wales Australia; 9 School of Allied Health Professionals Keele University Newcastle-under-Lyme, England United Kingdom; 10 NOSM University Thunder Bay, ON Canada; 11 School of Rehabilitation Science McMaster University Hamilton, ON Canada; 12 School of Nursing Faculty of Health Sciences Queen's University Kingston, ON Canada; 13 School of Physical and Occupational Therapy McGill University Montreal, QC Canada

**Keywords:** primary health care, clinical trial, health services, cluster randomized controlled trial, physiotherapy

## Abstract

**Background:**

Low back pain (LBP) is a common and disabling condition that is costly for health systems and society. Interprofessional primary care models may improve care quality and reduce this burden.

**Objective:**

This protocol and analysis plan communicates the methods for a cluster randomized trial with the following objectives: (1) evaluate the effectiveness of a physiotherapist-led (PT-led) primary care model for LBP at improving disability (primary outcome), pain intensity, quality of life, global rating of change, patient satisfaction, and adverse events compared with usual physician-led primary care; and (2) determine the impact of the PT-led primary care model for LBP on the health care system and society (health care access, health care use, missed work, cost-effectiveness). Both objectives are evaluated over a 1-year period. A multimethod process evaluation is embedded to assess model implementation, mechanisms, perspectives of patients and providers, and contextual influences.

**Methods:**

This study is a cluster randomized controlled trial with 20 primary care practices (clusters) in Canada, randomized 1:1 to a PT-led or usual physician-led primary care model for LBP. Adults seeking care from their primary care team for LBP are recruited over 1 year. Data collection occurs at baseline, 6 weeks, and 3, 6, 9, and 12 months. Effectiveness will be analyzed using linear mixed regression. The process evaluation analysis will include: descriptive and comparative analyses to assess implementation; descriptive and mediation analyses to assess potential mechanisms; qualitative interpretive description to understand experiences and perspectives of patients, PTs, and other health professionals; and mixed methods to determine contextual influences on implementation.

**Results:**

Recruitment of primary care sites (clusters) was completed in June 2023, following delays related to the COVID-19 pandemic. Cluster randomization occurred in July 2023. Recruitment of patient participants began in October 2023 and concluded in November 2024 (n=739). The final self-reported patient data was collected on November 25, 2025. Extraction of electronic health record data is scheduled for completion on December 19, 2025. Data analysis will be conducted in accordance with the study protocol and analysis plan and will begin once all data collection activities are complete. No interim analyses have been performed.

**Conclusions:**

The results of this trial will provide evidence for knowledge users to determine whether a PT-led primary care model for LBP is effective and should be adopted more widely. Knowledge users have identified the impact of the new model of care on disability, quality of life, and cost-effectiveness as key evidence needed to inform key decision-making. The multimethod process evaluation will provide critical evidence to interpret trial results and inform future scale and spread of this model of care if effective.

**Trial Registration:**

ClinicalTrials.gov NCT04287413; https://clinicaltrials.gov/study/NCT04287413

**International Registered Report Identifier (IRRID):**

DERR1-10.2196/89004

## Introduction

### Background

Low back pain (LBP) is the world’s largest contributor to years lived with disability [1], costs the Canadian health care system between CAD $6 and CAD $12 billion (US $4.4 to US $8.8 billion) annually [2], and is a leading contributor to lost work productivity [3,4]. The burden of LBP on the health care system is evidenced by frequent health care use, including unnecessary specialist consultations, diagnostic procedures [4,5], opioid prescriptions [6], and emergency department (ED) visits [7].

LBP is among the most common reasons for physician visits, with primary care physicians being the most frequent first point of contact within the health care system for people with LBP [8-10]. A *Lancet* [11-13] series on LBP highlighted international expert consensus on the need to evaluate new primary care models that better support physicians, who receive limited training in the management of musculoskeletal conditions and report low confidence addressing LBP [14-16]. One of these models involves integrating physiotherapists (PTs) within primary care teams at the first point of contact, which has the potential to provide patients with a more focused LBP consultation, assist with evidence-based treatment delivery, reduce low-value care (such as inappropriate imaging, specialist physician referrals, and opioid prescriptions), and reduce primary care physician visits. If integrating PTs can reduce primary care physician visits, it could contribute to increasing the capacity of primary care teams to help address the growing challenge of providing universal access to primary care [17] in the context of more complex patient encounters, increasing prevalence of multiple chronic conditions [18], and an aging population [19].

Evidence suggests PTs can provide collaborative care and implement recommendations from LBP primary care guidelines [20-23], including: screening for serious pathology, the need for diagnostics [24] and identifying risk factors for poor recovery [25]; providing reassurance, encouragement for early return to work, and exercise and physical activity recommendations [26]; and delivering targeted, psychologically informed interventions for those at risk of prolonged recovery [27]. Evidence from outside of Canada suggests that early guideline-adherent PT care for LBP improves function and disability [28,29] while reducing diagnostic imaging, opioid prescriptions, and physician specialist referrals [29-31], and reducing per-person health care costs [32,33].

Evidence from observational research suggests that involving PTs in providing first-point-of-contact care (ie, PT-led care) for those with work-related injuries in the US military resulted in workers being more satisfied with their care, receiving faster access to treatment, having fewer sickness absences, and using PTs and specialist physicians more appropriately [34-39]. Observational studies from the UK National Health Service on first-contact PT models of care indicate similar health outcomes, high levels of patient and physician satisfaction, shorter physician wait times, fewer work absences and diagnostic images, lower prescription medication use, and reduced costs [40-43].

The absence of high-quality randomized trials of PT-led primary care models leaves important unanswered questions about the process and impact of integrating PTs within primary care teams for people with LBP [44]. Specifically, there is a need for higher quality evidence on the impact of PT-led primary care on patient-oriented outcomes (eg, function, pain, and quality of life), health system outcomes (eg, health care access, physician workload, ED visits, specialist physician referrals, medication use, diagnostic imaging), and societal outcomes (eg, missed work, cost-effectiveness). Furthermore, it is unclear how PTs will navigate primary care challenges, such as providing care for people presenting with multiple health concerns or fulfilling requests for medications, diagnostic imaging, or notes for work absences. This cluster-randomized trial will address these gaps by assessing the impact of a PT-led primary care model for LBP. The results will inform primary care transformations across multiple health systems and potentially improve outcomes for patients with LBP.

The purpose of publishing this protocol and analysis plan is to transparently report our design and methods, and to transparently communicate our analytic plan in advance of completing data collection and carrying out our planned analysis to reduce the risk of analytic or reporting bias.

### Research Questions

The research questions involved in this study are as follows:

1. Is a PT-led primary care model for LBP effective at improving disability (primary outcome), pain intensity, quality of life, global rating of change (GROC), patient satisfaction, and adverse events compared with usual physician-led primary care, when evaluated over a 1-year period?
2. What is the impact of a PT-led primary care model for LBP on the health system and society (health care access, primary care physician workload, health care use, missed work, cost-effectiveness), evaluated over a 1-year period?

## Methods

### Trial Design

**Figure 1:**
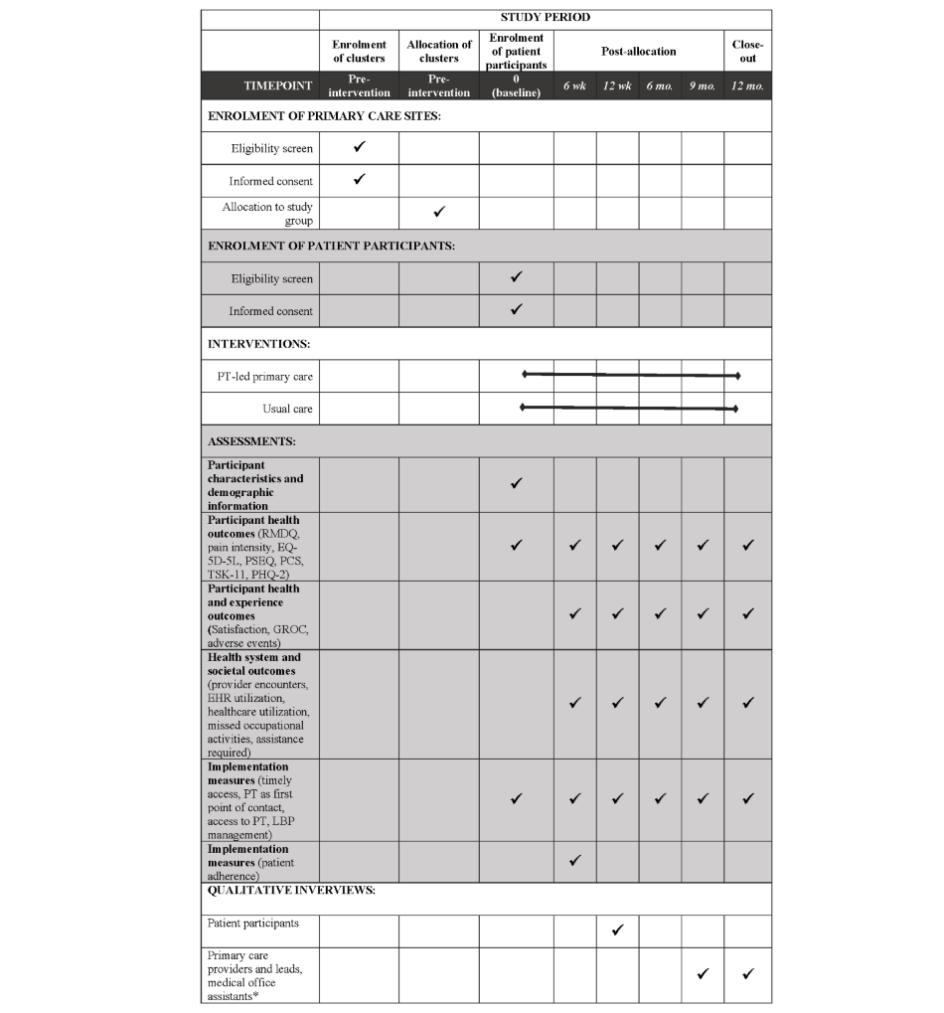
Schedule of enrolment, interventions, and assessments. EHR: electronic health record; EQ-5D-5L: EuroQOL-5D; GROC: global rating of change; LBP: low back pain; PCS: Pain Catastrophizing Scale; PHQ-2: 2-item Patient Health Questionnaire; PSEQ: Pain Self-Efficacy Questionnaire; PT: physiotherapist; RMDQ: Roland-Morris Disability Questionnaire; TSK-11: Tampa Scale of Kinesiophobia 11.

The trial is a parallel arm cluster randomized controlled trial at 20 primary care sites randomized 1:1 to a PT-led or usual physician-led primary care model for LBP. Randomization of practices, rather than patient participants, allows evaluation of a model where PTs are able to fully integrate within the primary care team and reduces potential contamination between study arms [45]. This protocol and analysis plan includes all of the items included in the SPIRIT (Standard Protocol Items: Recommendations for Interventional Trial) reporting guidelines [46,47]. Figure 1 provides a summary of the schedule of enrolment, interventions, and assessments.

**Figure 2:**
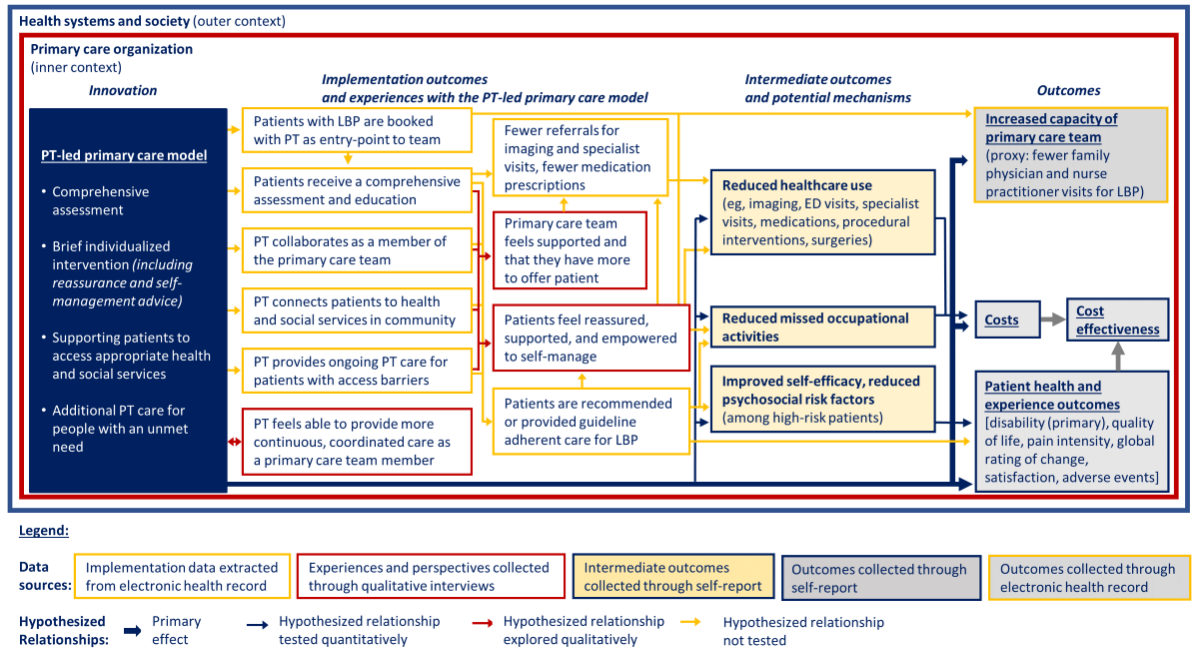
Physiotherapy-led primary care model program theory. LBP: low back pain; PT: physiotherapist.

The trial incorporates a mixed methods process evaluation informed by United Kingdom Medical Research Council guidance for developing and evaluating complex interventions [48,49], the Consolidated Framework for Implementation Research (CFIR) [50,51], and findings from the process evaluation from our pilot trial [52]. Process evaluations assess how interventions were implemented and under what conditions. They are important to help interpret trial results (eg, explaining why an intervention fails or has unexpected consequences, or why it works and how it can be refined). Process evaluations can be particularly valuable for informing future implementation and sustainability of complex health care interventions [49]. Our process evaluation is informed by and intended to refine our program theory [48] for the PT-led primary care model for LBP (Figure 2). The program theory describes how the PT-led primary care model for LBP is intended to lead to improvements in trial outcomes.

### Allocation of Participating Primary Care Teams to Trial Groups

Restricted randomization is recommended for cluster randomized trials to ensure the arms are balanced at baseline. We used covariate-constrained randomization [53] with a 1:1 ratio to the intervention and comparison arms to retain the merits of random allocation while ensuring baseline balance across the arms. We stratified by location (Southeastern Ontario or Interior British Columbia) and included the following covariates: number of active patients and rural versus urban setting. Our maximum tolerable difference in the rurality indicator was 1, and the strata balancing criteria for the number of active patients was set to 10%. An independent statistician implemented the procedure using a SAS macro [54]. Concealment was maintained by ensuring each practice had an anonymized code and performing randomization after all sites were recruited.

### Methods for Protecting Against Sources of Bias

Due to the trial design and interventions being compared, the PTs, patient participants, other members of the primary care team, and research assistants have not been blinded. While limitations due to the inability to blind are unavoidable, we have taken additional steps to minimize the risk of bias suggested for cluster trials [55]. A common challenge in cluster trials is identification and recruitment bias when patient recruitment must take place after cluster allocation [56]. To minimize the risks of these biases, we invited consecutive patients to participate, provided the same information about the trial to both groups prior to participants consenting (inviting participation in a study that involved either seeing their family physician as usual or seeing a PT added to their team in order not to reveal the cluster allocation until after consent and baseline data collection), employed research assistants unfamiliar with the patients to recruit and consent participants, and rigorously applied inclusion/exclusion criteria at all sites [57].

### Inclusion/Exclusion Criteria

For primary care sites, inclusion criteria were ≥2 family physicians or nurse practitioners and 1500 or more active patients. Exclusion criteria were: already having a PT on the team or no space available for a PT to practice. Patient participants were screened prior to consent by a trained research assistant at both intervention and comparison sites. Inclusion criteria for patient participants were: adults (≥19 years of age) with LBP of any duration, who were able to read, write, and speak English. Exclusion criteria were: known cancer that could possibly contribute to LBP, or inability to complete the scheduled follow-ups over 1 year.

### Recruitment

We recruited 20 sites (14 in Ontario and 6 in British Columbia, Canada). Site recruitment focused primarily on the engagement of contacted sites to reduce the risk of site withdrawal [58]. For patient participant recruitment, staff at each primary care site screened patients for their willingness to participate when they called to book an appointment. If patients booked via a web-based booking system, their reason for visit was screened by the clinic staff, and staff reached out to potential participants to ask about their willingness to be contacted by a research assistant. Trained research assistants screened potential participants using inclusion/exclusion criteria and enrolled consenting patients. Patients who raised LBP with any provider during a clinical encounter were also identified as potential participants. In these cases, they were directed to the administrative team, who then connected them with a research assistant for screening and enrollment. Balance in recruitment across arms and across clusters was monitored throughout the study, and strategies were implemented to maintain consistent implementation of recruitment processes across sites.

### Sample Size and Power Calculations

We used the methodology of Teerenstra et al [59] to calculate the required number of clusters based on an analysis of covariance for the primary outcome (Roland-Morris Disability Questionnaire [RMDQ]) at 12 months, adjusting for baseline. Our target sample was 20 clusters (10 per arm), allowing for 1560 patient participants (78 per practice before attrition). Conservatively accounting for attrition of 2 clusters (resulting in 18 clusters) and 20% of patients (resulting in 63 patients/cluster), this target would achieve 90% power to detect a minimally important mean difference of 2.5 points [60] (Cohen *d*=0.4) using a 2-sided α of 0.05 and assuming a SD of 5.7 points based on pilot study data, a conservative intracluster correlation coefficient of 0.1 [61], a cluster autocorrelation coefficient (correlation between cluster means at baseline and follow-up) of 0.5, and an individual autocorrelation coefficient (correlation between participant scores at baseline and follow-up) of 0.6 informed by our pilot study [62]. An average rate of 1.5 patient participants per site each week over 1 year would achieve the recruitment target of 1560 participants, which we anticipated based on our pilot study, where we recruited and retained 4 sites and achieved a recruitment rate of >1.7 participants/site/week. Recruitment of 400 patient participants across 18 clusters (22 participants/cluster) would achieve 80% power.

### Clinical Partner Sites

This study is being coordinated from a primary Research Coordinating Centre at Queen’s University with 20 participating primary care sites (clusters) between Southeastern Ontario (n=14) and Interior British Columbia (n=6), Canada. Sites include representation from urban and rural settings in both regions to facilitate generalizability.

### Patient and Public Involvement

A senior advisor for the Ontario Ministry of Health contributed to the plan for this study. An individual with lived experience is a coauthor on this protocol (LC) who has been engaged in the design of this study, including the identification and decision on the trial outcome measures that are important to patients. Their involvement in carrying out the study includes pretesting of data collection methods, interpretation of process evaluation results, and participation in the development of knowledge mobilization products (ie, designing summaries appropriate for patient organizations).

### Trial Interventions

#### Index Intervention: PT-Led Primary Care Model for LBP

##### Overview

The index intervention involves integrating a PT within the primary care team and making them available at the first point of contact for people with LBP. Patients with LBP are encouraged to book with the PT except when the primary reason for their visit is medication renewal or when additional health concerns require another provider’s attention. The intervention has four components: (1) assessment and screening; (2) brief individualized intervention; (3) supporting patients to access appropriate health services based on assessment findings; and (4) providing additional PT care to people with an unmet need. Ten registered PTs, who have completed 2 days of training on this care model, are participating in the delivery of the model across the 10 practices randomized to the PT-led primary care arm.

##### Assessment and Screening

The assessment and screening includes: taking a history; screening for pathology (eg, cauda equina syndrome, traumatic fracture, cancer); physical and neurological examination; application of evidence-based tools to identify comorbid health conditions (eg, 2-item Patient Health Questionnaire [PHQ-2] [63] for depression) requiring additional care; and using a validated tool (Keele STarT Back Tool [27,64]) to identify risk factors associated with persistent LBP and disability.

##### Brief Individualized Intervention

The PTs provide a brief individualized intervention at the initial visit. This intervention is intended to be based on primary care guidelines for LBP [23] and consists of effective communication to validate the patient’s experiences [65], cognitive reassurance [66], individually tailored exercises [67,68], and advice/strategies to stay active [69].

##### Supporting Patients to Access Appropriate Health Services Based on Assessment Findings

It is intended that the PT collaborates with the patient to identify appropriate health services based on assessment findings, collaborates with relevant primary care team members to provide the needed care, and integrates health service providers from outside of the team, considering the patient’s needs and access to health services. First, the PT identifies potential pathology needing urgent referral (eg, cauda equina, fracture, and infection). Next, they identify comorbid conditions that require collaboration with other primary care team members (eg, physicians, nurse practitioners, nurses, social workers, occupational therapists, pharmacists, and dieticians). For example, people who screen positive for depression are referred to their physician, nurse practitioner, or a member of the mental health team. Finally, primary care PTs refer to community PT as informed by the patient’s score on the Keele STarT Back tool [27,64] (if appropriate). The STarT Back tool categorizes patients with LBP into low, medium, or high risk of persistent pain and disability based on physical and psychosocial risk factors [64]. The recommended matched treatment for low-risk patients is to provide self-management advice and to avoid referral and investigations where possible. This intervention is brief, and it is expected that it can take place at the first visit. The recommended matched treatment for medium-risk patients is referral for standard community PT, and the matched treatment for high-risk patients is PT care from the primary care PT who received specific training aimed at reducing physical and psychosocial risk factors for chronic pain and disability as part of the 2-day training. This stratified approach to care has demonstrated improved function, quality of life, and cost-effectiveness in comparison to usual care in the United Kingdom [27]. When a need for PT care is identified, the primary care PT helps these patients navigate the available PT resources (ie, private PT clinics when the patient has private health insurance; government-funded PT for those who meet the criteria; or PT in primary care for those without access to a PT elsewhere).

##### Providing Additional PT Care to People With an Unmet Need

The PT provides additional care at the primary care site for patients who are appropriate for PT (based on STarT Back score) and who have barriers to accessing PT care in their community. For example, people who may benefit from physiotherapy but do not have access to services—due to a lack of private or government insurance coverage or because of geographic or transportation barriers—are offered additional physiotherapy care. Care includes evidence-based, guideline-consistent management, such as individualized education, exercise, and cognitive behavioral approaches. To avoid duplication of available PT services, patient participants with private- or government-funded health insurance for PT are referred to external PT services.

#### Comparison: Usual Physician-Led Care

The usual physician- or nurse practitioner-led primary care intervention has been unstandardized to reflect usual primary care clinical practice in Canada. Our pilot study suggested the most common management approaches in the usual care model included diagnostic imaging (12% of patients), medication renewal (21%), new medication prescription (14%), notes to employers (12%), and referral (PT 23%, chronic pain clinic 5%, massage therapy 5%, physician specialists 5%, and dietician 2%). Interventions provided or recommended by the physician have been and will continue to be recorded and monitored throughout the full trial.

There are no required or prohibited concomitant treatment options for either treatment arm. Should a participant choose to explore other or additional treatment options within their primary care team or from other health service providers, they are free to do so. This was made clear in the letter of information and consent form provided to the participants upon entry to the study.

### Duration of the Treatment Period

The PT-led primary care model is being implemented over a 1-year period from the time of consent. All participants were offered an initial assessment with the PT and have access to the PT for follow-up needs for a 1-year period after their initial assessment. The majority of participants classified as low-risk using the STarT Back tool, and those with private or government-funded health insurance for PT are intended to refine our program theory for the PT-led primary care model for LBP, but will have access to the PT as a member of their primary care team throughout the 1-year follow-up period if they need or want a follow-up visit. Participants identified as medium- or high-risk without access to PT elsewhere are offered additional PT care from the PT in primary care. The frequency and duration of the treatment plan is determined by the PT and the patient participant. In our pilot study, participants who took part in ongoing care received an average of 4 visits over 8 weeks.

### Intervention Modifications

We do not anticipate encountering a situation that would require the withdrawal of a participant for safety concerns related to the PT-led primary care or usual physician-led primary care. As per normal primary care and PT practice for LBP, the intervention will be modified by the primary care team to maintain participant safety as part of the model of care (eg, modification of exercise in response to increases in pain that may be experienced with an exercise intervention, discontinuation of medications if adverse effects are experienced). Due to the low-risk nature of the study and the fact that patient participants maintained access to their usual primary care providers, a Data Monitoring Committee was not established. Ongoing oversight and patient participant safety monitoring were provided by the study investigators, including monitoring all adverse events reported at all time points.

### Data Collection and Management

All patient participant-reported outcome measures are being collected from participants using REDCap (Research Electronic Data Capture; Vanderbilt University), a secure online survey and data capture tool hosted at Queen’s University [70,71]. A distinct link for the surveys is being sent to each participant at each assessment time point. We are providing the option of completing the questionnaires in person or by phone if participants have barriers to completing them online. Data are also being extracted from participants’ electronic health records (EHRs) related to care provided for LBP. Data are being extracted using prepiloted EHR extraction forms.

At study completion, responses to the surveys will be exported directly from REDCap to encrypted- and password-protected datasets and stored securely in Microsoft OneDrive at Queen’s University. All data collected from the EHR, along with a master linking log that links study identification numbers with participants, will be stored in password-protected and encrypted files in OneDrive. The linking log will be permanently destroyed at the end of the data analysis period. Qualitative interview recordings will be transcribed, deidentified, and stored securely in OneDrive.

We implemented multiple strategies to promote participant retention across all time points. Research assistants sent reminders every 2-3 days through personalized emails, phone calls, and text messages to encourage timely survey completion. When requested, surveys were completed in-person or by phone to strengthen engagement and minimize loss to follow-up.

### Frequency and Duration of Follow-Up

Patient characteristics and demographic information were collected at baseline. All patient-reported outcome measures are being collected at baseline, 6 weeks, and 3, 6, 9, and 12 months from the initial visit, with the primary comparison at 12 months for all outcomes. Patient satisfaction, GROC, adverse effects, and health care use are being collected at follow-up time points only. Patient adherence to PT recommendations is being collected at the 6-week follow-up.

### Participant Characteristics

#### Sociodemographic and Clinical Characteristics

The following characteristics and demographic information were collected from patients at baseline: age, first 3 digits of their postal code (to determine rural/urban status), biological sex, gender, identification as a member of a racialized group, duration of the current episode of LBP, previous history of LBP, number of other pain locations, highest level of education achieved, household income, and work status. In addition, the following questionnaires were administered at baseline as potential covariates and to inform subgroup analyses as part of our process evaluation.

#### Functional Comorbidity Index

An 18-item list of comorbidities that are associated with physical functioning. Each comorbidity is assigned a score of 1, and the total score is the sum of the comorbidity elements [72,73].

#### Keele STarT Back Tool

The Keele STarT Back Tool categorizes patients with LBP into low, medium, or high risk of persistent pain and disability based on physical and psychosocial risk factors [27,64,74].

### Patient Health and Experience Outcomes

#### Self-Reported Disability (Primary Outcome)

Self-reported disability (primary outcome) is measured using the RMDQ, which demonstrates reliability, validity, and responsiveness in people with acute and chronic LBP [75,76].

#### Pain Intensity

Pain intensity is measured using a Numeric Pain Rating Scale (0-10) [77] for pain at rest, pain when walking, and pain when lifting a bag of groceries from the floor. Each will be reported on a scale of 0 (no pain at all) to 10 (worst imaginable pain).

#### Health-Related Quality of Life

Health-related quality of life is assessed using the EuroQOL-5D (EQ-5D-5L) [78], which demonstrates good reliability and validity, and is suitable for economic evaluation in LBP [79]. The EQ-5D-5L Visual Analog Scale score (0-100) will be reported as a patient health outcome. The EQ-5D responses will be converted to an EQ-5D index value using the value set calculated for the Canadian context [80]. The index value will be reported as a patient-level health outcome and will also be used to calculate quality-adjusted life years (QALYs) for our outcome in the economic evaluation.

#### Global Rating of Change

Global Rating of Change is measured using an 11-point GROC scale that asks participants, “with respect to your functional abilities, how would you describe yourself now compared to how you were at your initial appointment for this episode of back pain?” Participants are asked to respond on a scale from a great deal better (+5) to a great deal worse (–5), as has been recommended for self-reported rating of change [81].

#### Participant Satisfaction

Participant satisfaction is measured using an 11-point scale for satisfaction with the care they received for their LBP, extremely dissatisfied (–5) to extremely satisfied (+5).

#### Adverse Events

Adverse events are assessed using a questionnaire consistent with reporting guidelines [82,83] that asks: (1) if the patient participant has experienced any adverse events as a result of any of the treatments received, (2) what adverse events were experienced, (3) how long the events lasted, and (4) how severe each adverse event was. For analysis, adverse events will be identified as serious or nonserious. An adverse event will be identified as serious if any of the following criteria are met: the participant requires inpatient hospitalization or an ED visit due to the adverse event, the adverse event results in significant and persistent disability (beyond 72 hours), or the adverse event is life-threatening or results in death. These responses are being monitored as they are completed in order to provide ongoing oversight of patient participant safety.

The following measures will be assessed as secondary outcomes, reported in the trial results, and included in the process evaluation as potential mechanisms through which the PT-led primary care model influences LBP-related disability.

#### Self-Efficacy

Self-efficacy is assessed as confidence in the ability to participate in usual activities using the Pain Self-Efficacy Questionnaire (PSEQ) [84,85]

#### Psychosocial Risk Factors for Persistent Pain and Disability

The Pain Catastrophizing Scale (PCS) [86,87], Tampa Scale of Kinesiophobia 11 (TSK-11) [88,89], and PHQ-2 [63,90] will measure psychosocial factors associated with pain-related disability.

### Health System and Societal Outcomes

#### Primary Care Physician or Nurse Practitioner Encounters

The number of new and repeat primary care physician or nurse practitioner visits for LBP per patient. This measure will be considered a proxy for a potential increase in primary care team capacity achieved if the LBP-related workload of primary care physicians or nurse practitioners is reduced, thus increasing their availability to provide care to other patients.

#### Health Care Usage Within the Primary Care Team

Health care usage within the primary care team is assessed using data collected from the EHR, including consultations with other primary care team members (eg, physicians, nurse practitioners, nurses, social workers, and occupational therapists) and group programming accessed within the primary care organization.

#### Health Care Use Outside of the Primary Care Team

Health care use outside of the primary care team is assessed using self-report data from follow-up surveys, cross-checked with EHR reports when possible, including medications used; walk-in clinic visits; ED visits; inpatient hospital stays; surgeries, injections, and other interventional procedures; visits to specialist physicians; diagnostic imaging; and visits to other health professionals outside the primary care team (eg, chiropractors, massage therapists, occupational therapists, PTs, chronic pain clinics).

#### Missed Occupational Activities

Missed occupational activities are assessed using self-report data from follow-up surveys, including time (days) lost from paid employment, volunteer, homemaking, or educational activities related to LBP.

#### Assistance Required

Assistance required is assessed using self-report data from follow-up surveys: paid and unpaid assistance required. For example, self-care (eg, taking medications, dressing/undressing, going to the bathroom, bathing/showering, grooming), shopping/groceries, meal preparation, housework, managing finances, or transportation (eg, to a medical appointment).

#### Costs

Total costs per person will be calculated by summing direct health care costs and indirect costs using a human capital approach for missed occupational activities.

#### Sources of Direct Health Care Cost Data

Intervention costs will include the PT salary and training needed to carry out the intervention. Costs for publicly funded health care services will be obtained from the Ontario Ministry of Health Schedule of Benefits [91]. Medication costs will be obtained from the Ontario Drug Benefit formulary. Expenses related to health services funded by private insurance or out of pocket will be collected through self-report at all follow-up assessments. Other costs incurred by the participant related to their LBP are also being collected by self-report, including support or assistance for self-care, housework, shopping, or transportation (eg, to health care appointments). The total direct costs will be determined by multiplying the quantity of resource use by the corresponding unit cost, summing the total cost over each follow-up interval, and then calculating the mean cost at each follow-up time point, as well as an overall mean cost for the entire study period.

#### Indirect Costs

Non-health care costs will be limited to loss of productivity using a human capital approach [92]. The mean provincial wage reported by Statistics Canada will be used to assign a monetary value to time lost from paid employment. The minimum wage value in Ontario and British Columbia will be used to place a value on time lost by those who were retired and time lost from volunteer, homemaking, caregiving, or educational activities.

#### Cost-Effectiveness

We will conduct a cost-utility analysis from both societal (primary) and health payer (secondary) perspectives to meet the needs of all knowledge users. For both societal and health payer perspectives, we will estimate the incremental cost-per-QALY gained [93-95].

### Implementation Measures

The following measures will be used to assess how the PT-led primary care model and the usual physician- or nurse practitioner-led primary care model were implemented.

#### Timely Access to LBP Care

Timely access to LBP care is assessed using the percentage of patient participants with LBP who are assessed within 48 hours of calling for an appointment. Participants who learned about the study and were invited to participate during an appointment for their LBP will not be included in this analysis.

#### PT as the First Point of Contact

Using the percentage of patient participants with LBP in the PT-led primary care arm who visited a PT as their first point of contact for the current episode of LBP.

#### Access to PT Services

PT as the first point of contact is assessed using the percentage of patients who are classified as medium- or high-risk on the STarT Back screening tool who access PT.

#### LBP Management

LBP management is described using data collected from the EHR, including education; exercise; psychological approaches; referrals to other primary care team members (eg, primary care physicians, nurse practitioners, nurses, social workers, occupational therapists, and group programming); referrals made to health professionals outside of the primary care team; medications prescribed, deprescribed, and suggested; diagnostic imaging ordered; lab work ordered and received; notes to employers or insurers; interprofessional communications with the primary care team; and other interventions provided.

#### Patient Adherence to Recommendations

Adherence to recommendations from the primary care PT is being collected at the 6-week follow-up. For patient participants identified as medium- or high-risk using the STarT Back classification, whether or not they accessed recommended PT (either through a referral to a community PT or through the PT in primary care) is being collected as part of our health use survey questions.

### Qualitative Interviews

Semistructured qualitative interviews are being conducted with patients, PTs, other health professionals, primary care team members, medical office assistants, and primary care organizational leaders. Interviews with patient participants, PTs, and other health professionals have two goals: (1) to explore the experiences and perspectives with the PT-led primary care model for LBP and (2) to understand how the model of care was implemented, how the intervention interacted with its context, and barriers/facilitators to implementation. Interview guides for interviews with patient participants, PTs, and other health professionals start by exploring experiences with the PT-led primary care model for LBP using open-ended questions and probing. The interview guides then focus on asking participants about how the model of care was implemented, how the intervention interacted with its context, and barriers/facilitators to implementation using questions constructed to align with the CFIR domains. The interview guides for medical office assistants and primary care organizational leaders will focus on how the model was implemented and contextual factors influencing implementation using the CFIR. Interview guides were prepiloted with persons with lived experience as patients and primary care team members prior to conducting the interviews.

We are using purposive sampling to recruit 8 to 12 patient participants with diversity in terms of age, gender, race, household income, LBP-related disability, LBP duration, STarT Back risk categories, and primary care site. We are inviting all PTs who are involved in implementing the PT-led primary care model for LBP to participate in an interview. We are purposively sampling 10 to 15 primary care health professionals who work with a PT in the PT-led primary care model for LBP, ensuring variation in terms of professional background, gender, and primary care site. We are using purposive sampling to identify 4 to 8 medical office assistants and 4 to 8 primary care organizational leaders who have experienced implementation of the PT-led primary care model. The concept of information power [96] related to our study objectives is being used to determine when to stop interviewing within each informant group based.

Patient participants were asked during their initial consent process for the main trial whether they were willing to be contacted for a follow-up qualitative interview about their experiences with the PT-led model of care. Willing patient participants were contacted within 12 weeks of enrollment. Based on our purposive sampling criteria, research assistants contacted potential participants who had agreed to be contacted to provide additional details about the interview purpose, review the consent process, and arrange a convenient time for the interview. Participants who agreed to take part were then sent a letter of information and a consent form in advance. At the time of the interview, the research assistant confirms that the participant has reviewed the consent form, responds to any questions, and obtains verbal consent before proceeding. Recruitment of health care providers, medical office assistants, and organizational leaders is being carried out by a study coordinator and research assistant who are familiar with the participating clinical teams. Potential nonpatient participants are being invited via email or in-person. Those who indicate interest are scheduled for an interview. As with patient participants, they will receive the letter of information and consent form ahead of the interview, and verbal consent is obtained at the start of the session after confirming their understanding and answering any questions.

### Protocol and Analysis Plan Amendments

Changes to the protocol and analysis plan will be communicated by amending the trial registry at ClinicalTrials.gov and reported in the full trial publication. Investigators and participants will be communicated with as appropriate based on the changes.

### Analysis

#### Effectiveness Analysis

All quantitative analyses will be conducted as per the Intention-To-Treat principle. Descriptive statistics for baseline characteristics and primary and secondary outcomes will be reported by arm using means (SD) or medians (IQR) for continuous variables and counts (percent) for categorical variables. All analyses will be performed in SAS (version 9.4; SAS Institute Inc). Differences between arms will be compared, accounting for site clustering using linear mixed models and generalized estimating equations (GEEs), and significance will be reported with *P* values.

#### Patient Health and Experience Outcomes

For our patient health outcomes, we will use linear mixed models to estimate individual patient participant outcomes adjusting for clustering by primary care center. Our primary outcome (RMDQ) with repeated measures at baseline and the 6-week, 3-, 6-, 9-, and 12-month follow-up time points will be analyzed using linear mixed regression (using PROC MIXED in SAS), estimated using restricted maximum likelihood estimation and a Kenward–Roger degrees of freedom correction [97] to account for a small number of clusters. The model will include fixed effects for time, intervention group by time interaction, (omitting the group main effect to ensure baseline differences are constrained to zero [98]), factors used in the covariate-constrained allocation procedure [99] (rurality of the cluster, number of active patients) and other prespecified covariates associated with LBP-related disability (patient participant age [100], sex [101], income [102], highest level of education achieved, duration of current episode of LBP [103], Functional Comorbidity Index score [100]). The correlation in repeated measures on the same participant will be modeled using a suitable covariance structure, identified using information criteria (Akaike and/or Bayesian information criteria). To account for clustering within practices, the site will be modeled as a random effect. The intervention effect will be obtained as the adjusted least square mean difference between arms at 12 months, with 95% CIs. Secondary comparisons will be obtained using least square mean differences at intermediate time points.

The use of restricted maximum likelihood estimation under an assumption of missing at random (MAR) allows the use of all available data without the need for multiple imputation. To examine the risk of bias due to missing data, we will compare the characteristics of those remaining and those lost to follow-up to identify factors associated with attrition. We will perform a sensitivity analysis for a missing not at random departure from our MAR assumption using a delta-adjusted imputation pattern mixture model [104,105] approach to investigate the robustness of our trial outcomes with regard to the missing values of the RMDQ. Within this sensitivity analysis approach, we will start with the posterior distributions suggested by an imputation model using multiple imputation by chained equations. Our imputation model will incorporate all variables in our primary analysis model, along with the last-observed-before-time covariates, and additional covariates needed for the pattern mixture. The sensitivity parameter (delta) will be introduced to explore how a departure from MAR affects results by specifying a maximum delta for each pattern of missing. We will set the maximum delta to be twice the residual sample SD from the observed data fit of the primary linear mixed model. Our sensitivity analysis will use multiple imputation 9 times to generate estimated treatment effects for a range of sensitivity [106].

Pain intensity, health-related quality of life (EQ-5D-5L), PSEQ, PCS, TSK-11, and PHQ-2 outcomes will be analyzed as described for the primary outcome, adjusted for the same covariates. Patient satisfaction and GROC outcomes have no baseline measures, and will be analyzed using ordinal logistic regression with clusters as random effects, adjusting for the same covariates as described above. When individual items are missing from within any of the questionnaires, we will use simple mean imputation as suggested by Chavance [106]. Serious adverse events will be presented descriptively by arm due to low expected counts. Any adverse events (yes/no) will be presented as incidence rates with CIs and compared by calculating relative risks with CIs from robust Poisson regression using GEE-type robust variance estimators (using PROC GLIMMIX with EMPIRICAL option in SAS) to account for clustering [107] and using an exchangeable working correlation matrix. We will use empirical covariance (“sandwich”) bias-adjusted (residual-based) estimators and the Fay and Graubard [108] correction to account for the small number of clusters in all models comparing incidence rates. In the case of nonconvergence or unstable estimates due to the small number of clusters, we would attempt to fit the model using an independent working correlation matrix. Other information related to nonserious adverse events (ie, severity and duration) will be reported descriptively.

We have planned a secondary analysis, a responder analysis [109], to compare the proportion of participants who experience a meaningful improvement in disability (RMDQ) in the PT-led primary care model arm versus the usual care arm. We will define a meaningful improvement as an improvement of greater than or equal to 30% improvement on the RMDQ, corresponding to an established minimally important difference among people with LBP [110,111]. We will calculate the proportion of participants who experience a meaningful improvement in each arm and compare between groups using robust Poisson regression, with GEE-type robust variance estimators to account for clustering [112]. We will use empirical covariance (“sandwich”) bias-adjusted (residual-based) estimators and the Fay and Graubard [108] correction to account for small number of clusters.

#### Health Care Use and Missed Occupational Activity Outcomes

For health care use and missed occupational activities outcomes, we will estimate the average effect at the patient level across the population (marginal models), accounting for clustering using GEE-type robust variance estimators and robust Poisson regression with an exchangeable working correlation matrix. All of the models generated for our health care use and missed occupational activities outcomes will include the same covariates as our analysis for patient health outcomes: age, sex, highest level of education achieved, income, duration of current episode of LBP, and Functional Comorbidity Index score (individual level), and primary care site rurality and number of active patients (cluster level). In the case of nonconvergence or model instability, a possibility for any binary outcomes with very low or high event rates given our small number of clusters [113], we will attempt to model outcomes using an independence working correlation matrix. If we continue to experience issues with nonconvergence or instability with alternate covariance structures, we will remove covariates, starting with duration of pain and income, based on theoretical grounds and existing evidence on the strength of relationships between our covariates and our outcomes.

Primary care physician or nurse practitioner visits will be presented as rates and compared using rate ratios with adjusted Poisson or negative binomial regression. Other health care use within the primary care team (whether there were any other consultations with interprofessional team members and whether or not there was group programming accessed), and health care use outside of the primary care team (medications, diagnostic imaging, walk-in clinic visits, ED visits, specialist physician visits, ED visits, hospital admissions, interventional procedures, surgeries, other health provider visits) will be presented as incidence rates and compared by calculating relative risks with CIs using robust Poisson regression, accounting for clustering [107]. These models will use time as an offset to account for variable follow-up times and will incorporate empirical covariance (“sandwich”) bias-adjusted (residual-based) estimators and the Fay and Graubard correction to account for a small number of clusters [108,114,115]. Time (days) lost from occupational activities (paid employment, volunteer, homemaking, or educational activities) and assistance required (hours of paid assistance, hours of unpaid assistance) due to LBP will be presented as rates and compared using rate ratios with negative binomial regression.

#### Economic Evaluation

We will estimate the cost-effectiveness of the PT-led care model from both a societal (primary) and health system payer (secondary) perspective to meet the needs of all knowledge users. The total costs will be determined by multiplying the quantity of resource use (or lost days) by the corresponding unit cost (or hourly wage), summing the total cost over each follow-up interval, and then calculating the mean cost at each follow-up time point, as well as an overall mean cost for the entire 1-year study period. Results will be presented as aggregated and disaggregated costs. Utility data will be generated using EQ-5D-5L index values (ie, utility scores) from all follow-up assessment time points. We will estimate QALYs for every participant, using the area under the curve approach, assuming linear interpolation between the measurements. To accommodate the hierarchical structure of the data, we will use bivariate multilevel modeling to estimate the incremental cost-effectiveness ratio using a calculation of cost-per-QALY gained for PT-led primary care versus usual care [93-95] and to estimate the incremental net benefit at various willingness-to-pay values. To account for clustering of study sites, as well as heterogeneity in costs and treatment effect across jurisdictions, we will model treatment group as a fixed effect and the study site as a random effect, adjusting for the same covariates as the primary analyses as fixed effects in our models. We will conduct a probabilistic sensitivity analysis using 10,000 Monte Carlo simulations to present the uncertainty in our cost-effectiveness estimates. Simulation results will be plotted on cost-effectiveness planes and we will generate cost-effectiveness acceptability curves to display the probability that the PT-led care model is cost-effective across a range of willingness to pay thresholds.

#### Subgroup Analyses

In alignment with Sex and Gender Equity in Research (SAGER) guidelines [116], we plan to conduct exploratory analyses for each of our effectiveness outcomes for males and females to explore potential sex differences in each of these outcomes. We will include sex and its interaction with time and group by time in the models. We will report the interaction *P* value along with forest plots to visualize the subgroup treatment effects, along with 95% CIs.

#### Process Evaluation Analysis

The multimethod process evaluation analysis will assess how the PT-led primary care model for LBP was implemented, the potential mechanisms of the model, the experiences and perspectives of patients and primary care team members toward the model, and how the context influenced implementation and outcomes. The analysis is guided by and intended to inform refinements to our program theory [48,117] for the PT-led primary care model for LBP (Figure 2).

We will assess how the PT-led primary care model and usual care model were implemented and how the model influenced health care for people with LBP by the following:

1. Describing and comparing the proportion of patient participants who received timely access to LBP care between trial arms. The intended implementation outcome is that patient participants seeking primary care for LBP receive timely access (within 48 hours) to an LBP assessment from a PT in the PT-led primary care model for LBP. Access within 48 hours will be presented as incidence rates with CIs and compared by calculating relative risks with CIs using GEE-type variance estimators to account for clustering [107], using an exchangeable working correlation matrix. We will use empirical covariance (“sandwich”) bias-adjusted (residual-based) estimators and the Fay and Graubard correction to account for a small number of clusters. We will incorporate the same covariates as with our effectiveness analysis.
2. Describing the proportion of patient participants in the PT-led primary care arm who saw the PT as the first point of contact for the current episode of LBP.
3. Describing and comparing the proportion of patient participants who are categorized as medium or high risk on the STarT Back tool who access PT services between trial arms. The intended implementation outcome is that patient participants with LBP at medium or high risk of ongoing pain and disability access PT services, either through a referral (for participants who have access to PT services) or through additional PT care from the primary care PT (for participants who would not otherwise have access to PT services). We will report the proportion of participants at medium or high risk receiving PT care as incidence rates with CIs and compared by calculating relative risks with CIs using GEE-type variance estimators, accounting for clustering [107], using an exchangeable working correlation matrix. We will use empirical covariance (“sandwich”) bias-adjusted (residual-based) estimators and the Fay and Graubard correction to account for small number of clusters [118-120]. We will incorporate the same covariates as with our effectiveness analysis.
4. Describing and comparing the LBP management provided between trial arms and comparing the care provided to practice guidelines using recent World Health Organization (WHO) LBP guidelines [121] to assess the alignment of the care provided with practice guideline recommendations. The 10 recommended interventions in the WHO guidelines are: structured education, exercise, needling therapies, spinal manipulation, massage, operant therapy, cognitive behavioral therapy, nonsteroidal anti-inflammatory drugs, topical cayenne pepper, and multicomponent biopsychosocial care. Interventions that are recommended against include: traction, ultrasound, transcutaneous electrical nerve stimulation, and lumbar supports. Additionally, referrals for diagnostic imaging or physician specialist visits for spinal injections or surgery consultations are rarely needed for LBP and thus will be compared between arms for a potential reduction in low-value care [122,123]. The intended implementation outcome is that patient participants receive care recommended in practice guidelines and do not receive care recommended against in practice guidelines, and that a low proportion of people receive referrals or prescriptions for diagnostic imaging, physician specialists, or medications outside of nonsteroidal anti-inflammatory drugs. Receipt of each intervention recommended by the guidelines, recommended against by the guidelines, and referrals (for imaging, spinal injections, or surgical consults) will be presented as incidence rates with CIs and compared by calculating relative risks with CIs using GEE-type robust variance estimates, accounting for clustering [107], using an exchangeable working correlation matrix. We will use empirical covariance (“sandwich”) bias-adjusted (residual-based) estimators and the Fay and Graubard correction to account for a small number of clusters [108]. We will incorporate the same covariates as with our effectiveness analysis.
5. Describing adherence to PT primary care recommendations. The intended implementation outcome is that patient participants report actioning the initial recommendations for physical activity and exercise, and that those who were recommended to access PT services report doing so.

To assess potential mechanisms of the PT-led primary care model, if there is a significant treatment effect, we will conduct a series of mediation analyses [124] to assess whether changes in LBP-related disability are explained by changes in self-efficacy (PSEQ) or changes in psychosocial risk factors (PHQ-2, PCS, TSK-11) for persistent LBP-related disability. We will carry out this mediation analysis for each of the self-efficacy and psychosocial risk factor variables in our entire sample and in the subgroup of patient participants identified as high-risk using the STarT Back classification. We hypothesize that change in self-efficacy will mediate the relationship between the intervention arm and RMDQ score in the full patient participant sample and that a change in psychosocial risk factors will mediate the relationship between the treatment arm and RMDQ score in the high-risk subgroup. We will use a stepwise approach to exploring the temporal trends and dynamics of the treatment effect across repeated measures as proposed by Beril et al [125], along with our theoretical insights regarding the mediation effect, when considering the appropriate mediation model. The indirect effect, or the intervention effect that can be explained by the mediator, will be determined as the difference between the total effect of the intervention and the direct effect of the intervention [126,127]. The significance of this effect can be used to statistically evaluate the possibility of mediation [128,129]. In this context, the total effect is the intervention effect obtained in the full model described in the trial. To be consistent with our primary outcome measure in the trial, we will be concerned with the time-specific mediation on the RMDQ outcome at the 12-month follow-up [130,131]. These effect measures will allow us to present the proportion of the total effect that is mediated through the respective measures of interest [132]. Development of the causal/associated conceptual model has allowed us to consider, and control for where needed, mediation analysis assumptions; that is, there is no intervention-outcome, mediator-outcome, or intervention-mediator confounding or mediator-outcome confounding that is influenced by the intervention itself [112,127,133]. Additionally, as part of exploring potential mechanisms, we will describe the proportion of any differences in total costs that are due to differences in health care use costs (and if so, what health care services) and what proportion of any differences in total costs are due to differences in costs due to missed occupational activities.

We will further explore potential mechanisms for intervention effects by conducting a planned subgroup analysis for all process outcomes, RMDQ, and costs based on STarT Back risk classification (low, medium, or high), recognizing that this analysis is exploratory and likely to be underpowered. We hypothesize that the participants in the PT-led group classified as low risk will be less likely to receive requisitions for diagnostic imaging, referrals to specialist physicians, and prescriptions for medications; demonstrate reduced costs; and show similar disability outcomes in comparison to patient participants in the usual care group. We hypothesize that participants in the PT-led group classified as medium risk will: be more likely to access PT services; receive LBP management closer aligned with guidelines; receive fewer requisitions for diagnostic imaging, prescriptions for medications, and referrals to specialist physicians; demonstrate reduced costs; and have reduced LBP-related disability. Finally, we hypothesize that participants in the PT-led group classified as high risk will: be more likely to access PT services; receive LBP management closer aligned with guidelines and more targeted care for psychosocial risk factors of persistent LBP-related disability; show greater reductions in the psychosocial risk factors; demonstrate reduced costs; and have reduced LBP-related disability.

To explore the experiences and perspectives of patients, PTs, and other primary care health professionals who have participated in the PT-led primary care model for LBP, qualitative interviews with patient participants, the PTs, and other health professional primary care team members will be recorded, transcribed, and coded independently by 2 investigators. An inductive qualitative analysis will be completed in an interpretive description tradition [118,119]. Interpretive description was chosen because of its emphasis on adding an interpretive lens during the analysis process in order identify meaningful themes from the data that can be applied in practice. This qualitative approach is therefore well aligned with our process evaluation goals to understand experiences and perspectives with the PT-led primary care model, potentially leading to refinements in the model of care, the program theory, or plans for scaling the intervention if effective. To promote rigor, we will use: 2 independent coders for the first 2 to 3 transcripts for each group (patient participants, PTs, other health professionals) and meet to reach agreement on the initial coding structure; reflexivity journaling and reflexive dialogue amongst team members throughout the analytic process; field notes and written memos; prolonged engagement within the data; and an audit trail of the research process and analytic decisions [120,134].

To explore how the context influenced the implementation of the PT-led primary care model for LBP, a concurrent mixed methods analysis will be conducted using the quantitative data collected to describe how the intervention is being implemented (analysis described above) and qualitative interview data from patient participants, PTs, other primary care health professionals, medical office assistants, and primary care organizational leaders. Qualitative analysis will begin by having multiple research team members immerse themselves in the data. Data will be coded by 2 independent research team members using an in-depth deductive (codes derived from CFIR constructs) and inductive (codes derived from the data) coding process in alignment with the CFIR User Guide [135] and previous research. Further, we will code relationships between constructs to capture how constructs interact, and how implementation determinants relate to how the model was implemented. Integration of qualitative and quantitative will be achieved through the design (convergent mixed methods), methods (merging), and interpretation (narrative and joint displays) [136]. Merging of qualitative and quantitative data will be achieved by linking participant characteristics, implementation data, and qualitative interview data. By bringing together the quantitative and qualitative data for analysis and linking this data at the level of the patient participant and cluster, we will be able to carry out an in-depth analysis of how context influenced implementation. Integration at the interpretation phase will be achieved using a weaving approach to interpret and report qualitative and quantitative findings together [136]. Joint displays will be used to bring data together in a visual display if appropriate.

### Ethical Considerations

Ethics approval for this study has been obtained from the Queen’s University Health Science and Affiliated Teaching Hospitals Research Ethics Board (HSREB #6027847). Written consent was obtained from all participants willing to participate. Research participants’ data was collected and stored securely to maintain privacy and confidentiality. Participants were provided with a CAD $10 (US $7.33) gift card for each completed assessment to thank them for their time.

### Knowledge Dissemination

We plan to mobilize the knowledge generated through this cluster randomized trial and process evaluation through a series of peer-reviewed manuscripts, with the following foci: (1) effectiveness of the PT-led primary care model for people with LBP (including one manuscript on patient health outcomes, and one on health system and societal outcomes), (2) how the PT-led primary care model for LBP was implemented, (3) potential mechanisms through which the PT-led primary care model for LBP influences patient health and health system outcomes (if the intervention is effective), (4) experiences and perspectives of patients with LBP who participated in the PT-led primary care model, (5) experiences and perspectives of PTs who participated in implementing the PT-led primary care model for people with LBP, (6) experiences and perspectives of other primary care team members who participated in implementing the PT-led primary care model for people with LBP, and (7) how the context influenced implementation of the PT-led primary care model for people with LBP. We plan to present these results at national and international conferences on primary care, health services, physiotherapy, and back pain. We will create tailored summary reports for each manuscript for each of the following knowledge user groups: patients, health professionals, primary care team leaders, and health system decision makers.

## Results

This trial was registered prospectively in January 2020 at ClinicalTrials.gov (NCT04287413). Recruitment of primary care sites (clusters) was completed in June 2023 (after being delayed due to the COVID-19 Pandemic). Cluster randomization was performed in July 2023. Patient participant recruitment was initiated in October 2023 and completed in November 2024. Last self-report patient data collected on November 25, 2025. EHR data extraction will be completed on December 19, 2025. Data analysis will be carried out in accordance with this protocol and analysis plan and will commence following the completion of data collection. No interim analyses have been conducted. Results are expected to be published in 2026.

## Discussion

The anticipated primary results of this trial are a robust estimate of the effectiveness of a PT-led primary care model on disability for people seeking primary care for LBP, as well as important estimates of the impact of the model of care on cost-effectiveness, health care use patterns, primary care team visits, and a number of secondary individual outcomes.

This cluster randomized trial will build on existing evidence by providing the most robust evidence to date on the effectiveness of a PT-led primary care model for people with LBP. A recent systematic review indicated that evidence on the effectiveness of PT-led primary care models on individual health outcomes and costs was limited to low or very low quality of evidence [137].

The strengths of this protocol are the pragmatic design and the detail in which we describe the methods and prespecified analysis plan to provide transparency and reproducibility of the methods and statistical considerations. Limitations of the study design include the fact that participant recruitment occurs after randomization of clinical sites, which prevents blinding of providers and may increase the risk of revealing allocation status to eligible patient participants, introducing a potential source of selection bias. Additionally, while the study is adequately powered to detect an effect for the primary outcome, the relatively small number of clusters may limit statistical power for secondary outcomes.

The results of this trial will inform new models of primary care in Canada and will be applicable to health systems around the world. Our detailed analysis plan has integrated the perspectives of knowledge users, including people living with LBP, primary care providers, PTs, researchers, and health system decision makers. These knowledge users have helped define study outcomes (ie, disability, quality of life, and cost-effectiveness) that will meet the needs of key knowledge users and decision makers. This protocol and analysis plan builds on our trial registration by articulating our analytic decisions in advance of analyzing our data to reduce the risk of analytic or reporting bias.

Our knowledge user team has also contributed to our detailed process evaluation, where we aim to explore how the PT-led primary care model for LBP was implemented, potential mechanisms of the model of care, experiences of people involved in implementation, and how context influences implementation. The updated Medical Research Council guidance for the development and evaluation of complex interventions emphasizes the importance of moving beyond only questions of effectiveness to also explore how complex interventions, like this model of care, will be accepted, adopted, implemented, scaled, and transferred across contexts [48]. Our analysis plan for an in-depth process evaluation aims to support health system decision makers by providing clear and transparent analytic processes to answer the key questions needed to inform the scale and spread of this model of care if it is effective.

## Appendix

Multimedia Appendix 1 Peer review documents from the Randomized Controlled Trials 2 Review Committee, Canadian Institutes of Health Research (CIHR).

## Abbreviations

CFIR: Consolidated Framework for Implementation Research
ED: emergency department
EHR: electronic health record
GEE: generalized estimating equation
GROC: global rating of change
LBP: low back pain
MAR: missing at random
PCS: Pain Catastrophizing Scale
PHQ-2: 2-item Patient Health Questionnaire
PSEQ: Pain Self-Efficacy Questionnaire
PT: physiotherapist
QALY: quality-adjusted life year
REDCap: Research Electronic Data Capture
RMDQ: Roland-Morris Disability Questionnaire
SAGER: Sex and Gender Equity in Research
SPIRIT: Standard Protocol Items: Recommendations for Interventional Trial
TSK-11: Tampa Scale of Kinesiophobia 11
WHO: World Health Organization

## Data Availability

The datasets generated and analyzed during this study are available from the corresponding author on reasonable request.

## References

[1] Vos T, Flaxman AD, Naghavi M, Lozano R, Michaud C, Ezzati M, Shibuya K, Salomon JA, Abdalla S, Aboyans V, Abraham J, Ackerman I, Aggarwal R, Ahn SY, Ali MK, Alvarado M, Anderson HR, Anderson LM, Andrews KG, Atkinson C, Baddour LM, Bahalim AN, Barker-Collo S, Barrero LH, Bartels DH, Basáñez M-G, Baxter A, Bell ML, Benjamin EJ, Bennett D, Bernabé E, Bhalla K, Bhandari B, Bikbov B, Bin Abdulhak A, Birbeck G, Black JA, Blencowe H, Blore JD, Blyth F, Bolliger I, Bonaventure A, Boufous S, Bourne R, Boussinesq M, Braithwaite T, Brayne C, Bridgett L, Brooker S, Brooks P, Brugha TS, Bryan-Hancock C, Bucello C, Buchbinder R, Buckle G, Budke CM, Burch M, Burney P, Burstein R, Calabria B, Campbell B, Canter CE, Carabin H, Carapetis J, Carmona L, Cella C, Charlson F, Chen H, Cheng AT, Chou D, Chugh SS, Coffeng LE, Colan SD, Colquhoun S, Colson KE, Condon J, Connor MD, Cooper LT, Corriere M, Cortinovis M, de Vaccaro KC, Couser W, Cowie BC, Criqui MH, Cross M, Dabhadkar KC, Dahiya M, Dahodwala N, Damsere-Derry J, Danaei G, Davis A, De Leo D, Degenhardt L, Dellavalle R, Delossantos A, Denenberg J, Derrett S, Des Jarlais DC, Dharmaratne SD, Dherani M, Diaz-Torne C, Dolk H, Dorsey ER, Driscoll T, Duber H, Ebel B, Edmond K, Elbaz A, Ali SE, Erskine H, Erwin PJ, Espindola P, Ewoigbokhan SE, Farzadfar F, Feigin V, Felson DT, Ferrari A, Ferri CP, Fèvre EM, Finucane MM, Flaxman S, Flood L, Foreman K, Forouzanfar MH, Fowkes FGR, Franklin R, Fransen M, Freeman MK, Gabbe BJ, Gabriel SE, Gakidou E, Ganatra HA, Garcia B, Gaspari F, Gillum RF, Gmel G, Gosselin R, Grainger R, Groeger J, Guillemin F, Gunnell D, Gupta R, Haagsma J, Hagan H, Halasa YA, Hall W, Haring D, Haro JM, Harrison JE, Havmoeller R, Hay RJ, Higashi H, Hill C, Hoen B, Hoffman H, Hotez PJ, Hoy D, Huang JJ, Ibeanusi SE, Jacobsen KH, James SL, Jarvis D, Jasrasaria R, Jayaraman S, Johns N, Jonas JB, Karthikeyan G, Kassebaum N, Kawakami N, Keren A, Khoo J, King CH, Knowlton LM, Kobusingye O, Koranteng A, Krishnamurthi R, Lalloo R, Laslett LL, Lathlean T, Leasher JL, Lee YY, Leigh J, Lim SS, Limb E, Lin JK, Lipnick M, Lipshultz SE, Liu W, Loane M, Ohno SL, Lyons R, Ma J, Mabweijano J, MacIntyre MF, Malekzadeh R, Mallinger L, Manivannan S, Marcenes W, March L, Margolis DJ, Marks GB, Marks R, Matsumori A, Matzopoulos R, Mayosi BM, McAnulty JH, McDermott MM, McGill N, McGrath J, Medina-Mora ME, Meltzer M, Mensah GA, Merriman TR, Meyer A, Miglioli V, Miller M, Miller TR, Mitchell PB, Mocumbi AO, Moffitt TE, Mokdad AA, Monasta L, Montico M, Moradi-Lakeh M, Moran A, Morawska L, Mori R, Murdoch ME, Mwaniki MK, Naidoo K, Nair MN, Naldi L, Narayan KMV, Nelson PK, Nelson RG, Nevitt MC, Newton CR, Nolte S, Norman P, Norman R, O'Donnell M, O'Hanlon S, Olives C, Omer SB, Ortblad K, Osborne R, Ozgediz D, Page A, Pahari B, Pandian JD, Rivero AP, Patten SB, Pearce N, Padilla RP, Perez-Ruiz F, Perico N, Pesudovs K, Phillips D, Phillips MR, Pierce K, Pion S, Polanczyk GV, Polinder S, Pope CA, Popova S, Porrini E, Pourmalek F, Prince M, Pullan RL, Ramaiah KD, Ranganathan D, Razavi H, Regan M, Rehm JT, Rein DB, Remuzzi G, Richardson K, Rivara FP, Roberts T, Robinson C, De Leòn FR, Ronfani L, Room R, Rosenfeld LC, Rushton L, Sacco RL, Saha S, Sampson U, Sanchez-Riera L, Sanman E, Schwebel DC, Scott JG, Segui-Gomez M, Shahraz S, Shepard DS, Shin H, Shivakoti R, Singh D, Singh GM, Singh JA, Singleton J, Sleet DA, Sliwa K, Smith E, Smith JL, Stapelberg NJC, Steer A, Steiner T, Stolk WA, Stovner LJ, Sudfeld C, Syed S, Tamburlini G, Tavakkoli M, Taylor HR, Taylor JA, Taylor WJ, Thomas B, Thomson WM, Thurston GD, Tleyjeh IM, Tonelli M, Towbin JA, Truelsen T, Tsilimbaris MK, Ubeda C, Undurraga EA, van der Werf MJ, van Os Jim, Vavilala MS, Venketasubramanian N, Wang M, Wang W, Watt K, Weatherall DJ, Weinstock MA, Weintraub R, Weisskopf MG, Weissman MM, White RA, Whiteford H, Wiersma ST, Wilkinson JD, Williams HC, Williams SRM, Witt E, Wolfe F, Woolf AD, Wulf S, Yeh P, Zaidi AKM, Zheng Z, Zonies D, Lopez AD, Murray CJL, AlMazroa MA, Memish ZA (2012). Years lived with disability (YLDs) for 1160 sequelae of 289 diseases and injuries 1990-2010: a systematic analysis for the global burden of disease study 2010. Lancet.

[2] (2022). Low back pain. Bone and Joint Canada.

[3] Dagenais S, Caro J, Haldeman S (2008). A systematic review of low back pain cost of illness studies in the United States and internationally. Spine J.

[4] Katz JN (2006). Lumbar disc disorders and low-back pain: socioeconomic factors and consequences. J Bone Joint Surg Am.

[5] Gandjour A, Telzerow A, Lauterbach KW, INTERCARE International Investigators (2005). European comparison of costs and quality in the treatment of acute back pain. Spine (Phila Pa 1976).

[6] Reid MC, Engles-Horton LL, Weber MB, Kerns RD, Rogers EL, O'Connor PG (2002). Use of opioid medications for chronic noncancer pain syndromes in primary care. J Gen Intern Med.

[7] Singer J (2011). A snapshot of health care in Canada as demonstrated by top 10 lists, 2011. Government of Canada Publications.

[8] Deyo RA, Mirza SK, Martin BI (2006). Back pain prevalence and visit rates: estimates from U.S. national surveys, 2002. Spine (Phila Pa 1976).

[9] Jordan KP, Kadam UT, Hayward R, Porcheret M, Young C, Croft P (2010). Annual consultation prevalence of regional musculoskeletal problems in primary care: an observational study. BMC Musculoskelet Disord.

[10] Côté P, Cassidy JD, Carroll L (2001). The treatment of neck and low back pain: who seeks care? who goes where?. Med Care.

[11] Buchbinder R, van Tulder M, Öberg B, Costa LM, Woolf A, Schoene M, Croft P, Lancet Low Back Pain Series Working Group (2018). Low back pain: a call for action. Lancet.

[12] Clark S, Horton R (2018). Low back pain: a major global challenge. Lancet.

[13] Foster NE, Anema JR, Cherkin D, Chou R, Cohen SP, Gross DP, Ferreira PH, Fritz JM, Koes BW, Peul W, Turner JA, Maher CG, Lancet Low Back Pain Series Working Group (2018). Prevention and treatment of low back pain: evidence, challenges, and promising directions. Lancet.

[14] Freedman KB, Bernstein J (2002). Educational deficiencies in musculoskeletal medicine. J Bone Joint Surg Am.

[15] Day CS, Yeh AC, Franko O, Ramirez M, Krupat E (2007). Musculoskeletal medicine: an assessment of the attitudes and knowledge of medical students at Harvard Medical School. Acad Med.

[16] Darlow B, Dean S, Perry M, Mathieson F, Baxter GD, Dowell A (2014). Acute low back pain management in general practice: uncertainty and conflicting certainties. Fam Pract.

[17] Petterson SM, Liaw WR, Phillips RL, Rabin DL, Meyers DS, Bazemore AW (2012). Projecting US primary care physician workforce needs: 2010-2025. Ann Fam Med.

[18] Barnett K, Mercer SW, Norbury M, Watt G, Wyke S, Guthrie B (2012). Epidemiology of multimorbidity and implications for health care, research, and medical education: a cross-sectional study. Lancet.

[19] Public Health Agency of Canada (2010). The chief public health officer's report on the state of public health in Canada 2010. Growing Older: Adding Life to Years.

[20] Delitto A, George SZ, Van Dillen L, Whitman JM, Sowa G, Shekelle P, Denninger TR, Godges JJ, Orthopaedic Section of the American Physical Therapy Association (2012). Low back pain. J Orthop Sports Phys Ther.

[21] Koes BW, van Tulder M, Lin CW, Macedo LG, McAuley J, Maher C (2010). An updated overview of clinical guidelines for the management of non-specific low back pain in primary care. Eur Spine J.

[22] Pillastrini P, Gardenghi I, Bonetti F, Capra F, Guccione A, Mugnai R, Violante FS (2012). An updated overview of clinical guidelines for chronic low back pain management in primary care. Joint Bone Spine.

[23] Low Back Pain Working Group (2017). Evidence-informed primary care management of low back pain - clinical practice guideline, 3rd edition. Institute of Health Economics.

[24] Comans T, Raymer M, O'Leary S, Smith D, Scuffham P (2014). Cost-effectiveness of a physiotherapist-led service for orthopaedic outpatients. J Health Serv Res Policy.

[25] Childs JD, Whitman JM, Sizer PS, Pugia ML, Flynn TW, Delitto A (2005). A description of physical therapists' knowledge in managing musculoskeletal conditions. BMC Musculoskelet Disord.

[26] Freburger JK, Carey TS, Holmes GM, Wallace AS, Castel LD, Darter JD, Jackman AM (2009). Exercise prescription for chronic back or neck pain: who prescribes it? who gets it? what is prescribed?. Arthritis Rheum.

[27] Hill JC, Whitehurst DGT, Lewis M, Bryan S, Dunn KM, Foster NE, Konstantinou K, Main CJ, Mason E, Somerville S, Sowden G, Vohora K, Hay EM (2011). Comparison of stratified primary care management for low back pain with current best practice (STarT Back): a randomised controlled trial. Lancet.

[28] Fritz JM, Cleland JA, Brennan GP (2007). Does adherence to the guideline recommendation for active treatments improve the quality of care for patients with acute low back pain delivered by physical therapists?. Med Care.

[29] Rutten GM, Degen S, Hendriks EJ, Braspenning JC, Harting J, Oostendorp RA (2010). Adherence to clinical practice guidelines for low back pain in physical therapy: do patients benefit?. Phys Ther.

[30] Riis A, Jensen CE, Bro F, Maindal HT, Petersen KD, Bendtsen MD, Jensen MB (2016). A multifaceted implementation strategy versus passive implementation of low back pain guidelines in general practice: a cluster randomised controlled trial. Implement Sci.

[31] Tahvonen P, Oikarinen H, Niinimäki J, Liukkonen E, Mattila S, Tervonen O (2017). Justification and active guideline implementation for spine radiography referrals in primary care. Acta Radiol.

[32] Fritz JM, Childs JD, Wainner RS, Flynn TW (2012). Primary care referral of patients with low back pain to physical therapy: impact on future health care utilization and costs. Spine (Phila Pa 1976).

[33] Childs JD, Fritz JM, Wu SS, Flynn TW, Wainner RS, Robertson EK, Kim FS, George SZ (2015). Implications of early and guideline adherent physical therapy for low back pain on utilization and costs. BMC Health Serv Res.

[34] Donato EB, DuVall RE, Godges JJ, Zimmerman GJ, Greathouse DG (2004). Practice analysis: defining the clinical practice of primary contact physical therapy. J Orthop Sports Phys Ther.

[35] Greathouse DG, Schreck RC, Benson CJ (1994). The United States army physical therapy experience: evaluation and treatment of patients with neuromusculoskeletal disorders. J Orthop Sports Phys Ther.

[36] James JJ, Stuart RB (1975). Expanded role for the physical therapist. screening musculoskeletal disorders. Phys Ther.

[37] McGill T (2013). Effectiveness of physical therapists serving as primary care musculoskeletal providers as compared to family practice providers in a deployed combat location: a retrospective medical chart review. Mil Med.

[38] Murphy BP, Greathouse D, Matsui I (2005). Primary care physical therapy practice models. J Orthop Sports Phys Ther.

[39] Ross MD, Childs JD, Middel C, Kujawa J, Brown D, Corrigan M, Parsons N (2014). Physical therapist vs. family practitioner knowledge of simple low back pain management in the U.S. Air Force. Mil Med.

[40] Walsh NE, Halls S, Thomas R, Berry A, Liddiard C, Cupples ME, Gage H, Jackson D, Cramp F, Stott H, Kersten P, Jagosh J, Foster D, Williams P (2024). First contact physiotherapy: an evaluation of clinical effectiveness and costs. Br J Gen Pract.

[41] Thompson J, Macintosh F, Beaumont N, Bedford L, Powley A, Bailey S (2024). The experiences and perceptions of first contact practitioners in primary care-a qualitative systematic review. Musculoskeletal Care.

[42] Lamb K, Comer C, Walsh N, McHugh G (2023). Patient access to first contact practitioner physiotherapists in the UK: a national survey. Musculoskeletal Care.

[43] Stynes S, Jordan KP, Hill JC, Wynne-Jones G, Cottrell E, Foster NE, Goodwin R, Bishop A (2021). Evaluation of the first contact physiotherapy (FCP) model of primary care: patient characteristics and outcomes. Physiotherapy.

[44] Docking S, Sridhar S, Haas R, Mao K, Ramsay H, Buchbinder R, O'Connor D (2025). Models of care for managing non-specific low back pain. Cochrane Database Syst Rev.

[45] Eccles M, Grimshaw J, Campbell M, Ramsay C (2003). Research designs for studies evaluating the effectiveness of change and improvement strategies. Qual Saf Health Care.

[46] Chan A, Tetzlaff JM, Gøtzsche PC, Altman DG, Mann H, Berlin JA, Dickersin K, Hróbjartsson A, Schulz KF, Parulekar WR, Krleza-Jeric K, Laupacis A, Moher D (2013). SPIRIT 2013 explanation and elaboration: guidance for protocols of clinical trials. BMJ.

[47] Chan AW, Boutron I, Hopewell S, Moher D, Schulz KF, Collins GS, Tunn R, Aggarwal R, Berkwits M, Berlin JA, Bhandari N, Butcher NJ, Campbell MK, Chidebe RCW, Elbourne DR, Farmer AJ, Fergusson DA, Golub RM, Goodman SN, Hoffmann TC, Ioannidis JPA, Kahan BC, Knowles RL, Lamb SE, Lewis S, Loder E, Offringa M, Ravaud P, Richards DP, Rockhold FW, Schriger DL, Siegfried NL, Staniszewska S, Taylor RS, Thabane L, Torgerson DJ, Vohra S, White IR, Hróbjartsson A (2025). SPIRIT 2025 statement: updated guideline for protocols of randomized trials. Nat Med.

[48] Skivington K, Matthews L, Simpson SA, Craig P, Baird J, Blazeby JM, Boyd KA, Craig N, French DP, McIntosh E, Petticrew M, Rycroft-Malone J, White M, Moore L (2021). A new framework for developing and evaluating complex interventions: update of medical research council guidance. BMJ.

[49] Moore GF, Audrey S, Barker M, Bond L, Bonell C, Hardeman W, Moore L, O'Cathain A, Tinati T, Wight D, Baird J (2015). Process evaluation of complex interventions: medical research council guidance. BMJ.

[50] Damschroder LJ, Reardon CM, Widerquist MAO, Lowery J (2022). The updated consolidated framework for implementation research based on user feedback. Implement Sci.

[51] Damschroder LJ, Reardon CM, Opra Widerquist MA, Lowery J (2022). Conceptualizing outcomes for use with the consolidated framework for implementation research (CFIR): the CFIR outcomes addendum. Implement Sci.

[52] Vader K, Donnelly C, French SD, Grady C, Hill JC, Tripp DA, Williams A, Miller J (2022). Implementing a new physiotherapist-led primary care model for low back pain: a qualitative study of patient and primary care team perspectives. BMC Prim Care.

[53] Moulton LH (2004). Covariate-based constrained randomization of group-randomized trials. Clin Trials.

[54] Chaudhary MA, Moulton LH (2006). A SAS macro for constrained randomization of group-randomized designs. Comput Methods Programs Biomed.

[55] Puffer S, Torgerson D, Watson J (2003). Evidence for risk of bias in cluster randomised trials: review of recent trials published in three general medical journals. BMJ.

[56] Campbell MJ (2014). Challenges of cluster randomized trials. J Comp Eff Res.

[57] Eldridge S, Kerry S, Torgerson DJ (2009). Bias in identifying and recruiting participants in cluster randomised trials: what can be done?. BMJ.

[58] Flynn TN, Whitley E, Peters TJ (2002). Recruitment strategies in a cluster randomized trial--cost implications. Stat Med.

[59] Teerenstra S, Eldridge S, Graff M, de Hoop E, Borm GF (2012). A simple sample size formula for analysis of covariance in cluster randomized trials. Stat Med.

[60] Bombardier C, Hayden J, Beaton DE (2001). Minimal clinically important difference. low back pain: outcome measures. J Rheumatol.

[61] Campbell MK, Fayers PM, Grimshaw JM (2005). Determinants of the intracluster correlation coefficient in cluster randomized trials: the case of implementation research. Clin Trials.

[62] Miller J, Barber D, Donnelly C, French S, Green M, Hill J, MacDermid J, Marsh J, Norman K, Richardson J, Taljaard M, Wideman T, Cooper L, McPhee C (2017). Determining the impact of a new physiotherapist-led primary care model for back pain: protocol for a pilot cluster randomized controlled trial. Trials.

[63] Thombs BD, Benedetti A, Kloda LA, Levis B, Nicolau I, Cuijpers P, Gilbody S, Ioannidis JPA, McMillan D, Patten SB, Shrier I, Steele RJ, Ziegelstein RC (2014). The diagnostic accuracy of the patient health questionnaire-2 (PHQ-2), patient health questionnaire-8 (PHQ-8), and patient health questionnaire-9 (PHQ-9) for detecting major depression: protocol for a systematic review and individual patient data meta-analyses. Syst Rev.

[64] Hill JC, Dunn KM, Main CJ, Hay EM (2010). Subgrouping low back pain: a comparison of the STarT back tool with the orebro musculoskeletal pain screening questionnaire. Eur J Pain.

[65] Linton SJ (2015). Intricacies of good communication in the context of pain: does validation reinforce disclosure?. Pain.

[66] Pincus T, Holt N, Vogel S, Underwood M, Savage R, Walsh DA, Taylor SJC (2013). Cognitive and affective reassurance and patient outcomes in primary care: a systematic review. Pain.

[67] Gordon R, Bloxham S (2016). A systematic review of the effects of exercise and physical activity on non-specific chronic low back pain. Healthcare (Basel).

[68] Searle A, Spink M, Ho A, Chuter V (2015). Exercise interventions for the treatment of chronic low back pain: a systematic review and meta-analysis of randomised controlled trials. Clin Rehabil.

[69] Dahm KT, Brurberg KG, Jamtvedt G, Hagen KB (2010). Advice to rest in bed versus advice to stay active for acute low-back pain and sciatica. Cochrane Database Syst Rev.

[70] Harris PA, Taylor R, Minor BL, Elliott V, Fernandez M, O'Neal L, McLeod L, Delacqua G, Delacqua F, Kirby J, Duda SN, REDCap Consortium (2019). The REDCap consortium: building an international community of software platform partners. J Biomed Inform.

[71] Harris PA, Taylor R, Thielke R, Payne J, Gonzalez N, Conde JG (2009). Research electronic data capture (REDCap)--a metadata-driven methodology and workflow process for providing translational research informatics support. J Biomed Inform.

[72] Groll DL, To T, Bombardier C, Wright JG (2005). The development of a comorbidity index with physical function as the outcome. J Clin Epidemiol.

[73] Rundell SD, Resnik L, Heagerty PJ, Kumar A, Jarvik JG (2020). Performance of the functional comorbidity index (FCI) in prognostic models for risk adjustment in patients with back pain. PM R.

[74] Main CJ, Sowden G, Hill JC, Watson PJ, Hay EM (2012). Integrating physical and psychological approaches to treatment in low back pain: the development and content of the STarT back trial's 'high-risk' intervention (StarT back; ISRCTN 37113406). Physiotherapy.

[75] Chiarotto A, Maxwell LJ, Terwee CB, Wells GA, Tugwell P, Ostelo RW (2016). Roland-morris disability questionnaire and oswestry disability index: which has better measurement properties for measuring physical functioning in nonspecific low back pain? systematic review and meta-analysis. Phys Ther.

[76] Roland M, Fairbank J (2000). The Roland-Morris disability questionnaire and the oswestry disability questionnaire. Spine (Phila Pa 1976).

[77] Haefeli M, Elfering A (2006). Pain assessment. Eur Spine J.

[78] van RM, Janssen B, Stolk E, Boye KS, Herdman M, Kennedy-Martin M (2019). EQ-5D-5L user guide. EuroQol.

[79] Finch AP, Dritsaki M, Jommi C (2016). Generic preference-based measures for low back pain: which of them should be used?. Spine (Phila Pa 1976).

[80] Xie F, Pullenayegum E, Gaebel K, Bansback N, Bryan S, Ohinmaa A, Poissant L, Johnson JA, Canadian EQ-5D-5L Valuation Study Group (2016). A time trade-off-derived value set of the EQ-5D-5L for Canada. Med Care.

[81] Kamper SJ, Maher CG, Mackay G (2009). Global rating of change scales: a review of strengths and weaknesses and considerations for design. J Man Manip Ther.

[82] Eldridge SM, Chan CL, Campbell MJ, Bond CM, Hopewell S, Thabane L, Lancaster GA, PAFS consensus group (2016). CONSORT 2010 statement: extension to randomised pilot and feasibility trials. Pilot Feasibility Stud.

[83] Ioannidis JPA, Evans SJW, Gøtzsche Peter C, O'Neill RT, Altman DG, Schulz K, Moher D, CONSORT Group (2004). Better reporting of harms in randomized trials: an extension of the CONSORT statement. Ann Intern Med.

[84] Chiarotto A, Vanti C, Cedraschi C, Ferrari S, de Lima E Sà Resende F, Ostelo RW, Pillastrini P (2016). Responsiveness and minimal important change of the pain self-efficacy questionnaire and short forms in patients with chronic low back pain. J Pain.

[85] Nicholas MK (2007). The pain self-efficacy questionnaire: taking pain into account. Eur J Pain.

[86] Osman A, Barrios FX, Kopper BA, Hauptmann W, Jones J, O'Neill E (1997). Factor structure, reliability, and validity of the pain catastrophizing scale. J Behav Med.

[87] Sullivan MJ, Thorn B, Haythornthwaite JA, Keefe F, Martin M, Bradley LA, Lefebvre JC (2001). Theoretical perspectives on the relation between catastrophizing and pain. Clin J Pain.

[88] Roelofs J, Goubert L, Peters ML, Vlaeyen JWS, Crombez G (2004). The Tampa scale for kinesiophobia: further examination of psychometric properties in patients with chronic low back pain and fibromyalgia. Eur J Pain.

[89] Swinkels-Meewisse EJCM, Swinkels RAHM, Verbeek ALM, Vlaeyen JWS, Oostendorp RAB (2003). Psychometric properties of the Tampa scale for kinesiophobia and the fear-avoidance beliefs questionnaire in acute low back pain. Man Ther.

[90] Manea L, Gilbody S, McMillan D (2015). A diagnostic meta-analysis of the patient health questionnaire-9 (PHQ-9) algorithm scoring method as a screen for depression. Gen Hosp Psychiatry.

[91] NA OHIP schedule of benefits and fees. Government of Ontario.

[92] Drummond M, Scupher M, Claxton K, Stoddard G, Torrance G (2005). Methods for the Economic Evaluation of Health Care Programs.

[93] Gomes M, Grieve R, Nixon R, Edmunds WJ (2012). Statistical methods for cost-effectiveness analyses that use data from cluster randomized trials: a systematic review and checklist for critical appraisal. Med Decis Making.

[94] Gomes M, Ng ES, Grieve R, Nixon R, Carpenter J, Thompson SG (2012). Developing appropriate methods for cost-effectiveness analysis of cluster randomized trials. Med Decis Making.

[95] Grieve R, Nixon R, Thompson SG (2010). Bayesian hierarchical models for cost-effectiveness analyses that use data from cluster randomized trials. Med Decis Making.

[96] Malterud K, Siersma VD, Guassora AD (2016). Sample size in qualitative interview studies: guided by information power. Qual Health Res.

[97] Kenward MG, Roger JH (2009). An improved approximation to the precision of fixed effects from restricted maximum likelihood. Comput Stat Data Anal.

[98] Hooper R, Forbes A, Hemming K, Takeda A, Beresford L (2018). Analysis of cluster randomised trials with an assessment of outcome at baseline. BMJ.

[99] Hemming K, Taljaard M (2023). Key considerations for designing, conducting and analysing a cluster randomized trial. Int J Epidemiol.

[100] Shmagel A, Foley R, Ibrahim H (2016). Epidemiology of chronic low back pain in US adults: data from the 2009-2010 national health and nutrition examination survey. Arthritis Care Res (Hoboken).

[101] George SZ, Fritz JM, Childs JD, Brennan GP (2006). Sex differences in predictors of outcome in selected physical therapy interventions for acute low back pain. J Orthop Sports Phys Ther.

[102] Mathieu J, Roy K, Robert ME, Akeblersane M, Descarreaux M, Marchand AA (2024). Sociodemographic determinants of health inequities in low back pain: a narrative review. Front Public Health.

[103] Bekkering GE, Hendriks HJM, van Tulder MW, Knol DL, Simmonds MJ, Oostendorp RAB, Bouter LM (2005). Prognostic factors for low back pain in patients referred for physiotherapy: comparing outcomes and varying modeling techniques. Spine (Phila Pa 1976).

[104] Moreno-Betancur M, Chavance M (2016). Sensitivity analysis of incomplete longitudinal data departing from the missing at random assumption: methodology and application in a clinical trial with drop-outs. Stat Methods Med Res.

[105] Troendle JF, Sur A, Leifer ES, Powell-Wiley T (2025). Sensitivity analyses for missing in repeatedly measured outcome data. Stat Med.

[106] Chavance M (2004). Handling missing items in quality of life studies. Commun Stat Theory Methods.

[107] Yelland LN, Salter AB, Ryan P (2011). Performance of the modified poisson regression approach for estimating relative risks from clustered prospective data. Am J Epidemiol.

[108] Fay M, Graubard B (2001). Small-sample adjustments for wald-type tests using sandwich estimators. Biometrics.

[109] Henschke N, van Enst A, Froud R, Ostelo RWG (2014). Responder analyses in randomised controlled trials for chronic low back pain: an overview of currently used methods. Eur Spine J.

[110] Dworkin RH, Turk DC, Wyrwich KW, Beaton D, Cleeland CS, Farrar JT, Haythornthwaite JA, Jensen MP, Kerns RD, Ader DN, Brandenburg N, Burke LB, Cella D, Chandler J, Cowan P, Dimitrova R, Dionne R, Hertz S, Jadad AR, Katz NP, Kehlet H, Kramer LD, Manning DC, McCormick C, McDermott MP, McQuay HJ, Patel S, Porter L, Quessy S, Rappaport BA, Rauschkolb C, Revicki DA, Rothman M, Schmader KE, Stacey BR, Stauffer JW, von Stein T, White RE, Witter J, Zavisic S (2008). Interpreting the clinical importance of treatment outcomes in chronic pain clinical trials: IMMPACT recommendations. J Pain.

[111] Ostelo RWJG, Deyo RA, Stratford P, Waddell G, Croft P, Von Korff M, Bouter LM, de Vet HC (2008). Interpreting change scores for pain and functional status in low back pain: towards international consensus regarding minimal important change. Spine (Phila Pa 1976).

[112] VanderWeele TJ (2010). Bias formulas for sensitivity analysis for direct and indirect effects. Epidemiology.

[113] Qiu H, Cook AJ, Bobb JF (2024). Evaluating tests for cluster-randomized trials with few clusters under generalized linear mixed models with covariate adjustment: a simulation study. Stat Med.

[114] Li P, Redden DT (2015). Small sample performance of bias-corrected sandwich estimators for cluster-randomized trials with binary outcomes. Stat Med.

[115] Thompson JA, Leyrat C, Fielding KL, Hayes RJ (2022). Cluster randomised trials with a binary outcome and a small number of clusters: comparison of individual and cluster level analysis method. BMC Med Res Methodol.

[116] Heidari S, Babor TF, De Castro P, Tort S, Curno M (2016). Sex and gender equity in research: rationale for the SAGER guidelines and recommended use. Res Integr Peer Rev.

[117] Funnell SC, Rogers PJ (2011). Purposeful Program Theory: Effective Use of Theories of Change and Logic Models.

[118] Hunt MR (2009). Strengths and challenges in the use of interpretive description: reflections arising from a study of the moral experience of health professionals in humanitarian work. Qual Health Res.

[119] Thorne S, Kirkham SR, MacDonald-Emes J (1997). Interpretive description: a noncategorical qualitative alternative for developing nursing knowledge. Res Nurs Health.

[120] Morse JM, Barrett M, Mayan M, Olson K, Spiers J (2002). Verification strategies for establishing reliability and validity in qualitative research. Int J Qual Methods.

[121] O'Sullivan K (2024). Appraisal of clinical practice guideline: World Health Organization guideline for non-surgical management of chronic primary low back pain in adults in primary and community care settings. J Physiother.

[122] Buchbinder R, Underwood M, Hartvigsen J, Maher CG (2020). The lancet series call to action to reduce low value care for low back pain: an update. Pain.

[123] Hartvigsen J, Kamper SJ, French SD (2022). Low-value care in musculoskeletal health care: is there a way forward?. Pain Pract.

[124] Hayes AF (2017). Introduction to Mediation, Moderation, and Conditional Process Analysis: A Regression Based Approach.

[125] Berli C, Inauen J, Stadler G, Scholz U, Shrout PE (2021). Understanding between-person interventions with time-intensive longitudinal outcome data: longitudinal mediation analyses. Ann Behav Med.

[126] VanderWeele TJ (2013). A three-way decomposition of a total effect into direct, indirect, and interactive effects. Epidemiology.

[127] VanderWeele TJ (2016). Mediation analysis: a practitioner's guide. Annu Rev Public Health.

[128] Zhao X, Lynch JG, Chen Q (2010). Reconsidering Baron and Kenny: myths and truths about mediation analysis. J Consum Res.

[129] Preacher KJ (2015). Advances in mediation analysis: a survey and synthesis of new developments. Annu Rev Psychol.

[130] Selig JP, Preacher KJ (2009). Mediation models for longitudinal data in developmental research. Res Hum Dev.

[131] Goldsmith KA, MacKinnon DP, Chalder T, White PD, Sharpe M, Pickles A (2018). Tutorial: the practical application of longitudinal structural equation mediation models in clinical trials. Psychol Methods.

[132] Ananth CV (2019). Proportion mediated in a causal mediation analysis: how useful is this measure?. BJOG.

[133] Richiardi L, Bellocco R, Zugna D (2013). Mediation analysis in epidemiology: methods, interpretation and bias. Int J Epidemiol.

[134] Holloway I, Galvin K (2016). Qualitative Research in Nursing and Healthcare.

[135] Reardon CM, Damschroder LJ, Ashcraft LE, Kerins C, Bachrach RL, Nevedal AL, Domlyn AM, Dodge J, Chinman M, Rogal S (2025). The consolidated framework for implementation research (CFIR) user guide: a five-step guide for conducting implementation research using the framework. Implement Sci.

[136] Fetters MD, Curry LA, Creswell JW (2013). Achieving integration in mixed methods designs-principles and practices. Health Serv Res.

[137] Abuhl B, Ehrmantraut D, Wolden M (2025). First-contact physical therapy compared to usual primary care for musculoskeletal disorders: a systematic review and meta-analysis of randomized controlled trials. Phys Ther.

