# Supplementary material for: Determining the Impact of a Physiotherapist-Led Primary Care Model for Low Back Pain: Protocol and Analysis Plan for a Cluster Randomized Controlled Trial and Embedded Process Evaluation
Source: JMIR Res Protoc. 2026 Feb 26;15:e89004. doi: 10.2196/89004 (PMC12982963; doi:10.2196/89004)
Supplement: Multimedia Appendix 1 [file resprot_v15i1e89004_app1.pdf]

July/Juillet 10, 2019

DC0190GP

Canadian Institutes of Health Research / Instituts de recherche en santé du Canada

Notice of Decision / Avis de décision

Application Number/Numéro de la demande: 419558

Committee Code/Code du comité: RC2

Applicants/Candidats: Dr. Jordan Miller

With/Avec:				
	Dr. D. Barber	Ms. L. Cooper	Dr. C. Donnelly	Dr. S. French
	Dr. M. Green	Dr. J. Hill	Dr. J. Macdermid	Dr. J. Marsh
	Mrs. C. McPhee	Dr. K. Norman	Dr. J. Richardson	Dr. G. Salisbury
	Dr. M. Taljaard	Dr. J. Whittaker	Dr. T. Wideman	

Institution paid/ Établissement payé: Queen's University (Kingston, Ontario)

Title/Titre: Determining the impact of a physiotherapist-led primary care model for low back pain - A cluster randomized controlled trial

Primary Inst./ Inst. principal: Health Services and Policy Research / Services et politiques de la santé

Other Related Inst./ Autres inst. connexes: Musculoskeletal Health and Arthritis / Appareil locomoteur et arthrite

Competition Outcome/Résultats du concours: Project Grant / Subvention Projet

March/Mars 06, 2019

Number in competition/Nbre de demandes dans le concours: 2445

Number approved/Nbre de demandes approuvées: 382

Decision on your application/ Décision sur votre demande: Approved / Approuvée

Total Funding Amount:/ Montant total du financement: $1,403,776

Term/Durée: 4 **yrs/ans** 0 **months/mois**

Peer Review Committee Recommendation, for your information and use/
Recommandation du comité d'examen par les pairs, pour fins d'information et d'utilisation:

**Committee/Comité:**	Randomized Controlled Trials 2 / Essais contrôlés randomisés 2
**Number reviewed/ Nbre de demandes examinées:**	37
**Number approved in that committee/ Nbre de demandes approuvées dans ce comité:**	5
**Application rank within the committee/ Rang de la demande dans ce comité:**	8
**Percent Rank Within the Committee/ Rang en pourcentage au sein du comité:**	80.56%
**Rating/ Cote:**	4.18

Additional Funding Opportunities/ Opportunités de financement additionnelles	Decision/ Décision	Total Funding Amount/Montant total du financement	Competition Code/Cote de concours	Application Number/ Numéro de la demande
Project Grant - PA: MSK, Skin and Oral Health: Clinical Research - MSK Health/Subv. Projet - AP: Santé muscul./cutanée/buccod.: Rech. clinique - santé muscul.	Not Approved/ Non approuvée	$0	201903PJ5	422920

This document is for information only. Official payment is stated on the CIHR Authorization for Funding.
Document à titre d'information seulement. Le paiement officiel est indiqué sur l'autorisation de financement des IRSC.

Canadian Institutes of Health Research
160 Elgin Street, 9th Floor
Address Locator 4809A
Ottawa, Ontario K1A 0W9

Instituts de recherche en santé du Canada
160, rue Elgin, 9e étage
Indice de l'adresse 4809A
Ottawa (Ontario) K1A 0W9

Institute of Aging
Institute of Cancer Research
Institute of Circulatory and Respiratory Health
Institute of Gender and Health
Institute of Genetics
Institute of Health Services and Policy Research
Institute of Human Development and Child and Youth Health
Institute of Indigenous Peoples' Health
Institute of Infection and Immunity
Institute of Musculoskeletal Health and Arthritis
Institute of Neurosciences, Mental Health and Addiction
Institute of Nutrition, Metabolism and Diabetes
Institute of Population and Public Health

Institut du vieillissement
Institut du cancer
Institut de la santé circulatoire et respiratoire
Institut de la santé des femmes et des hommes
Institut de génétique
Institut des services et des politiques de la santé
Institut du développement et de la santé des enfants et des adolescents
Institut de la santé des Autochtones
Institut des maladies infectieuses et immunitaires
Institut de l'appareil locomoteur et de l'arthrite
Institut des neurosciences, de la santé mentale et des toxicomanies
Institut de la nutrition, du métabolisme et du diabète
Institut de la santé publique et des populations

July 10, 2019

Dr. Jordan Miller
School of Rehabilitation Therapy
Louise D. Action Building
31 George Street
Queen's University
Kingston, Ontario K7L 3N6

Dear Dr. Miller,

On behalf of the Canadian Institutes of Health Research (CIHR), I am pleased to inform you that your application entitled “Determining the impact of a physiotherapist-led primary care model for low back pain - A cluster randomized controlled trial”, submitted to the Project Grant: Spring 2019 competition, has been approved for funding.

CIHR is committed to equalizing the success rate for Early Career Researchers (ECRs) to ensure that the proportion of ECRs funded equals the proportion of ECR applicants to the Project Grant competition. As part of this commitment, your application was determined eligible to receive funding.

Your application reviews and competition results are available through ResearchNet. If you are unable to view these documents, please contact us at support-soutien@cihr-irsc.gc.ca. Your Authorization for Funding will follow in the mail.

As CIHR does not notify co-applicants of the decision, we ask that you inform those individuals involved, along with their research institutions (if different from your own) of the outcome of this application.

Should you have any questions, please do not hesitate to communicate with a Processing Officer in the Contact Centre at 613-954-1968 or by e-mail at support-soutien@cihr-irsc.gc.ca.

Congratulations on your success in this competition.

Sincerely,

Martine Lafrance

Martine Lafrance, Ph.D.
Manager, Program Design and Delivery
Research Programs Portfolio

486057-201903PJT-RC2-419558-211432-PJNIR

Canadian Institutes of Health Research
160 Elgin Street, 9th Floor
Address Locator 4809A
Ottawa, Ontario K1A 0W9

Instituts de recherche en santé du Canada
160, rue Elgin, 9e étage
Indice de l'adresse 4809A
Ottawa (Ontario) K1A 0W9

Institute of Aging
Institute of Cancer Research
Institute of Circulatory and Respiratory Health
Institute of Gender and Health
Institute of Genetics
Institute of Health Services and Policy Research
Institute of Human Development and Child and Youth Health
Institute of Indigenous Peoples' Health
Institute of Infection and Immunity
Institute of Musculoskeletal Health and Arthritis
Institute of Neurosciences, Mental Health and Addiction
Institute of Nutrition, Metabolism and Diabetes
Institute of Population and Public Health

Institut du vieillissement
Institut du cancer
Institut de la santé circulatoire et respiratoire
Institut de la santé des femmes et des hommes
Institut de génétique
Institut des services et des politiques de la santé
Institut du développement et de la santé des enfants et des adolescents
Institut de la santé des Autochtones
Institut des maladies infectieuses et immunitaires
Institut de l'appareil locomoteur et de l'arthrite
Institut des neurosciences, de la santé mentale et des toxicomanies
Institut de la nutrition, du métabolisme et du diabète
Institut de la santé publique et des populations

July 10, 2019

Dr. Jordan Miller
School of Rehabilitation Therapy
Louise D. Action Building
31 George Street
Queen's University
Kingston, Ontario K7L 3N6

Dear Dr. Miller:

On behalf of the Canadian Institutes of Health Research (CIHR), I wish to congratulate you on your success in the recent CIHR funding competition!

As health researchers, we are united by a common goal: to improve the health and well-being of Canadians and people throughout the world. Through CIHR, the Government of Canada provides vital support to Canadian researchers, spanning the tightly linked pillars of health research, with the ultimate goal of improving health for all Canadians. As a recipient of this funding, you are now part of this endeavour.

As you are aware, the evaluation of your grant application was made possible thanks to peer reviewers who generously volunteer their time to support the Canadian health research enterprise. As a CIHR-funded researcher, your knowledge and expertise are invaluable to this process. If you are not already a peer reviewer, I would like to take this opportunity to invite you to consider becoming a member of the College of Reviewers. Please visit www.cihr-irsc.gc.ca/e/49923.html to learn about the eligibility criteria and how to apply for membership.

As health researchers, we are living at a time when science is under careful scrutiny, and it has become more important than ever to ensure that our work is clearly understood. As such, I would ask that you recognize your CIHR funding when delivering presentations and communicating about your research, and that you continue to advocate for the critical importance of research in advancing the health of Canadians.

Once again, please accept my congratulations on this impressive achievement and my best wishes for success in all your endeavours. I look forward to following the progress of your research.

Sincerely,

Michael J. Strong, MD, FRCPC, FAAN, FCAHS
President

485788-201903PJT-RC2-419558-211432-CONGR

Canada

CIHR IRSC
Discoveries for life / Découvertes pour la vie

**Review Type / Type d'évaluation:**	Reviewer 1 / Évaluateur 1
**Name of Applicant / Nom du chercheur:**	Miller, Jordan
**Application No. / Numéro de demande:**	419558
**Agency / Agence:**	CIHR/IRSC
**Competition / Concours:**	Project Grant/Subvention Projet
**Committee / Comité:**	Randomized Controlled Trials 2/Essais contrôlés randomisés 2
**Title / Titre:**	Determining the impact of a physiotherapist-led primary care model for low back pain - A cluster randomized controlled trial

Adjudication Criteria/Critères de sélection

Significance and Impact of the Research/Importance et impact de la recherche: 4.3

Approaches and Methods/Approches et méthodes: 4.3

Expertise, Experience and Resources/Expertise, expérience et ressources: 4.4

Top/Bottom Selection/Groupe supérieur/inférieur

- (●) **Top/Groupe supérieur**
- ( ) **Bottom/Groupe inférieur**

**Review Type / Type d'évaluation:**	Reviewer 1 / Évaluateur 1
**Name of Applicant / Nom du chercheur:**	Miller, Jordan
**Application No. / Numéro de demande:**	419558
**Agency / Agence:**	CIHR/IRSC
**Competition / Concours:**	Project Grant/Subvention Projet
**Committee / Comité:**	Randomized Controlled Trials 2/Essais contrôlés randomisés 2
**Title / Titre:**	Determining the impact of a physiotherapist-led primary care model for low back pain - A cluster randomized controlled trial

Summary of Application/Résumé de la demande:

Resubmission by an early career investigator. The applicants are proposing a 1560-patient, 20-primary care practices cluster randomized controlled trial of PT-led primary care versus physician-led care in patients with low back pain of any duration in 2 Ontario regions in both urban and rural settings. The primary outcome is self-reported disability. Follow-up is 12 months. An economic evaluation is built in.

The applicants have addressed previous reviewers' comments adequately, particularly since their pilot trial now has 12-month follow-up, they account for PT clustering in their analysis, have recruited sites in another region

**Review Type / Type d'évaluation:**	Reviewer 1 / Évaluateur 1
**Name of Applicant / Nom du chercheur:**	Miller, Jordan
**Application No. / Numéro de demande:**	419558
**Agency / Agence:**	CIHR/IRSC
**Competition / Concours:**	Project Grant/Subvention Projet
**Committee / Comité:**	Randomized Controlled Trials 2/Essais contrôlés randomisés 2
**Title / Titre:**	Determining the impact of a physiotherapist-led primary care model for low back pain - A cluster randomized controlled trial

Strengths and Weaknesses/Forces et faiblesses:

STRENGTHS:

- The grant is very well written
- An important health problem is addressed, the applicants build a strong case.
- Pilot data available to inform on the feasibility with a published protocol and a manuscript under review
- A cost-effectiveness analysis is included
- Multidisciplinary research team with complementary expertise
- Engagement of stakeholders including patients.
- Good strategies to minimize loss to follow-up including an option for in-person or on the phone final follow-up.

WEAKNESSES:

- Only 3 PTs in the PT-led group, affecting generalizability.

**Review Type / Type d'évaluation:**	Reviewer 1 / Évaluateur 1
**Name of Applicant / Nom du chercheur:**	Miller, Jordan
**Application No. / Numéro de demande:**	419558
**Agency / Agence:**	CIHR/IRSC
**Competition / Concours:**	Project Grant/Subvention Projet
**Committee / Comité:**	Randomized Controlled Trials 2/Essais contrôlés randomisés 2
**Title / Titre:**	Determining the impact of a physiotherapist-led primary care model for low back pain - A cluster randomized controlled trial

Budget Recommendation/Recommandation budgétaire:

no concern

Please indicate your appraisal of the integration of sex as a biological variable as a strength, weakness, or not applicable to the proposal./Prière de sélectionner une option pour donner votre évaluation de l'intégration du sexe comme variable biologique en tant que point fort ou point faible de la proposition, ou en tant qu'élément non applicable à la proposition.

- ◉ **Strength/Point fort**
- ◯ **Weakness/Point faible**
- ◯ **Not applicable/Non applicable**

Please indicate your appraisal of the integration of gender as a socio-cultural determinant of health as a strength, weakness, or not applicable to the proposal./Prière de sélectionner une option pour donner votre évaluation de l'intégration du genre comme déterminant socioculturel de la santé en tant que point fort ou point faible de la proposition, ou en tant qu'élément non applicable à la proposition.

- ◉ **Strength/Point fort**
- ◯ **Weakness/Point faible**
- ◯ **Not applicable/Non applicable**

Sex and/or Gender Considerations/Notions de sexe et/ou de genre:

The applicant team includes a sex-gender chair. A subgroup analysis by sex is pre-specified. The importance of that subgroup analysis is well justified.

**Review Type / Type d'évaluation:**	Reviewer 2 / Évaluateur 2
**Name of Applicant / Nom du chercheur:**	Miller, Jordan
**Application No. / Numéro de demande:**	419558
**Agency / Agence:**	CIHR/IRSC
**Competition / Concours:**	Project Grant/Subvention Projet
**Committee / Comité:**	Randomized Controlled Trials 2/Essais contrôlés randomisés 2
**Title / Titre:**	Determining the impact of a physiotherapist-led primary care model for low back pain - A cluster randomized controlled trial

Adjudication Criteria/Critères de sélection

Significance and Impact of the Research/Importance et impact de la recherche: 4.2

Approaches and Methods/Approches et méthodes: 3.8

Expertise, Experience and Resources/Expertise, expérience et ressources: 4.0

Top/Bottom Selection/Groupe supérieur/inférieur

- ◉ **Top/Groupe supérieur**
- ◯ **Bottom/Groupe inférieur**

**Review Type / Type d'évaluation:**	Reviewer 2 / Évaluateur 2
**Name of Applicant / Nom du chercheur:**	Miller, Jordan
**Application No. / Numéro de demande:**	419558
**Agency / Agence:**	CIHR/IRSC
**Competition / Concours:**	Project Grant/Subvention Projet
**Committee / Comité:**	Randomized Controlled Trials 2/Essais contrôlés randomisés 2
**Title / Titre:**	Determining the impact of a physiotherapist-led primary care model for low back pain - A cluster randomized controlled trial

Summary of Application/Résumé de la demande:

This application will explore the impact of integrating a physiotherapist (PT) within the primary care team for people with low back pain (LBP). This is a cluster-randomized trial including 20 sites and 1560 patients. PT will be involved in four components: 1) Primary care assessment and screening; 2) Brief PT intervention for all people during their initial visit; 3) Collaboration with primary care team and identification of appropriate health services; 4) Providing additional PT care to people with an unmet need. Primary outcome is function over a one-year timeframe, with a variety of secondary outcomes including health economics and process evaluation.

**Review Type / Type d'évaluation:**	Reviewer 2 / Évaluateur 2
**Name of Applicant / Nom du chercheur:**	Miller, Jordan
**Application No. / Numéro de demande:**	419558
**Agency / Agence:**	CIHR/IRSC
**Competition / Concours:**	Project Grant/Subvention Projet
**Committee / Comité:**	Randomized Controlled Trials 2/Essais contrôlés randomisés 2
**Title / Titre:**	Determining the impact of a physiotherapist-led primary care model for low back pain - A cluster randomized controlled trial

Strengths and Weaknesses/Forces et faiblesses:

Significance and Impact of the Research

Low back pain (LBP) is extremely common in Canada and a leading cause of morbidity, with a huge cost to the healthcare system in direct costs and lost productivity. Conventional management of LBP in the primary care model has been largely ineffective. There is a strong need for better approaches to this common problem.

There have been several proposed models for managing LBP in primary care, one of which involves integrative physiotherapists (PT) as the first point of care. It is not clear why this model was chosen, or whether this represents the most promising approach. There are many other members of the multidisciplinary team with a focus on LBP; were they consulted or involved in this proposal?

There have been no randomized trials to date examining this question. This trial has the potential to provide evidence of the impact of PT involvement in LBP that could be immediately integrated into the healthcare system, with the potential for real improvement in patient outcome and savings to the system.

The trial also has the potential to reduce prescription for opioids, which would be a hugely important outcome given the current opioid crisis. This could be incorporated as another secondary outcome.

The applicants have incorporated the opinions of patients, primary-care providers, PTs, researchers, and policy-makers. This is a strength.

More details of the knowledge translation plan would strengthen the application.

Approaches and Methods

The applicants have chosen a cluster-randomized design to reduce potential contamination from PTs and family physicians concurrently managing intervention and control patients. This is a significant concern and well-addressed by this design.

There are several evaluations focused on implementation and health-systems effects of the intervention, including a qualitative component and process modelling. This strengthens the study considerably.

The trial will be limited to three PTs who have completed seven days of training on this care model. These PTs will be available for same-day or next-day appointments. These specialized practitioners and immediate availability seem contrary to the otherwise pragmatic design of this trial and limit the applicability of the trial findings. The trial would be strengthened by inclusion of more PTs, widely representative of what could be found in practice in Canada.

Overall there seems to be variability within the trial in terms of pragmatic vs explanatory elements. For example, the PT arm is very specialized and the protocol for managing patients is very specific, however the standard of care arm is completely open to the practitioner's discretion. The trial would be stronger if these elements were aligned – either all pragmatic or all explanatory based on the applicants' intentions. The background suggests that the trial results will be directly implemented which implies a pragmatic design, but the actual trial design does not follow this assumption.

The trial is limited to Ontario; it would be strengthened by including sites from other parts of the Country. There are significant variations in patient characteristics and system factors across the country and these need to be accounted for in a trial like this if the results are to be implemented broadly.

The applicants conducted a pilot trial that initially demonstrated differential enrollment at intervention vs control sites. Strategies were implemented to address this and for the remaining pilot trial enrollment were balanced. This is a major strength of the application but will need to be monitored closely on an ongoing basis to reduce recruitment bias.

Expertise, Experience and Resources

A pilot trial was conducted that demonstrated the feasibility of this approach, although it included only one PT and four sites. The enrollment rate is likely to be lower at the additional sites so will need to be monitored closely. But the overall approach is feasible.

The trial will be coordinated by the same coordinator who led the pilot trial, supported a methods center. This should be a feasible approach. There are some risks to having three RAs supporting all 20 sites across the province, when consent is needed from individual participants. Travel could make this challenging.

The trial steering committee will consist of four members who are independent of trial investigators, and the study Principal Investigator will not be included. This seems unusual; what is the rationale for this?

**Review Type / Type d'évaluation:**	Reviewer 2 / Évaluateur 2
**Name of Applicant / Nom du chercheur:**	Miller, Jordan
**Application No. / Numéro de demande:**	419558
**Agency / Agence:**	CIHR/IRSC
**Competition / Concours:**	Project Grant/Subvention Projet
**Committee / Comité:**	Randomized Controlled Trials 2/Essais contrôlés randomisés 2
**Title / Titre:**	Determining the impact of a physiotherapist-led primary care model for low back pain - A cluster randomized controlled trial

Budget Recommendation/Recommandation budgétaire:

Budget seems reasonable for a trial of this scope.

Please indicate your appraisal of the integration of sex as a biological variable as a strength, weakness, or not applicable to the proposal./Prière de sélectionner une option pour donner votre évaluation de l'intégration du sexe comme variable biologique en tant que point fort ou point faible de la proposition, ou en tant qu'élément non applicable à la proposition.

- ◉ **Strength/Point fort**
- ○ **Weakness/Point faible**
- ○ **Not applicable/Non applicable**

Please indicate your appraisal of the integration of gender as a socio-cultural determinant of health as a strength, weakness, or not applicable to the proposal./Prière de sélectionner une option pour donner votre évaluation de l'intégration du genre comme déterminant socioculturel de la santé en tant que point fort ou point faible de la proposition, ou en tant qu'élément non applicable à la proposition.

- ◉ **Strength/Point fort**
- ○ **Weakness/Point faible**
- ○ **Not applicable/Non applicable**

**Review Type / Type d'évaluation:**	Reviewer 2 / Évaluateur 2
**Name of Applicant / Nom du chercheur:**	Miller, Jordan
**Application No. / Numéro de demande:**	419558
**Agency / Agence:**	CIHR/IRSC
**Competition / Concours:**	Project Grant/Subvention Projet
**Committee / Comité:**	Randomized Controlled Trials 2/Essais contrôlés randomisés 2
**Title / Titre:**	Determining the impact of a physiotherapist-led primary care model for low back pain - A cluster randomized controlled trial

Sex and/or Gender Considerations/Notions de sexe et/ou de genre:

The study will record sex and gender identity and the applicants will use this data to conduct a series of secondary analyses to determine the influence of sex and gender on the three objectives of the trial (determining the clinical effectiveness, health system impact, and processes of a new PT-led primary care model for back pain in comparison to usual physician led care) by considering sex as a covariate and by using disaggregated analysis. The applicants will also monitor enrollment to ensure they recruit adequate representation of men and women in the full trial and the qualitative interviews.

**Review Type / Type d'évaluation:**	Reviewer 3 / Évaluateur 3
**Name of Applicant / Nom du chercheur:**	Miller, Jordan
**Application No. / Numéro de demande:**	419558
**Agency / Agence:**	CIHR/IRSC
**Competition / Concours:**	Project Grant/Subvention Projet
**Committee / Comité:**	Randomized Controlled Trials 2/Essais contrôlés randomisés 2
**Title / Titre:**	Determining the impact of a physiotherapist-led primary care model for low back pain - A cluster randomized controlled trial

Adjudication Criteria/Critères de sélection

Significance and Impact of the Research/Importance et impact de la recherche: 4.3

Approaches and Methods/Approches et méthodes: 4.3

Expertise, Experience and Resources/Expertise, expérience et ressources: 4.4

Top/Bottom Selection/Groupe supérieur/inférieur

◉ **Top/Groupe supérieur**
○ **Bottom/Groupe inférieur**

**Review Type / Type d'évaluation:**	Reviewer 3 / Évaluateur 3
**Name of Applicant / Nom du chercheur:**	Miller, Jordan
**Application No. / Numéro de demande:**	419558
**Agency / Agence:**	CIHR/IRSC
**Competition / Concours:**	Project Grant/Subvention Projet
**Committee / Comité:**	Randomized Controlled Trials 2/Essais contrôlés randomisés 2
**Title / Titre:**	Determining the impact of a physiotherapist-led primary care model for low back pain - A cluster randomized controlled trial

Summary of Application/Résumé de la demande:

The applicants propose a 20-site (1560 pts) cluster randomized clinical trial comparing physiotherapist-led care for back pain vs. conventional physician-led care. Outcomes include clinical (pain scores, functional measures, etc.) quality of life, AEs, etc. at BL, 6w, 3m, 6m, 9m, 12m.

**Review Type / Type d'évaluation:**	Reviewer 3 / Évaluateur 3
**Name of Applicant / Nom du chercheur:**	Miller, Jordan
**Application No. / Numéro de demande:**	419558
**Agency / Agence:**	CIHR/IRSC
**Competition / Concours:**	Project Grant/Subvention Projet
**Committee / Comité:**	Randomized Controlled Trials 2/Essais contrôlés randomisés 2
**Title / Titre:**	Determining the impact of a physiotherapist-led primary care model for low back pain - A cluster randomized controlled trial

Strengths and Weaknesses/Forces et faiblesses:

Strengths

+ Experienced team well able to carry out the work.

+ Clinical problem with large potential for improved patient care and savings to health care systems.

+ Cluster-randomized design appropriate, planned analyses broadly appropriate (except for niggling concern regarding impact of covariate-constrained randomization, additional detail regarding handling of repeated-measures outcomes would be appropriate).

+ Apparent "buy-in" by all sites planned to be recruited.

Weaknesses

- Assessments are not (could not be?) blinded - the pt and the caregivers all know which group they are in. With soft endpoints, this could be seriously problematic and the trial design provides no opportunity to assess this. Corroboration of soft endpoint outcomes with strong (expensive) hard endpoints in a subset of pts at all sites might be able to help with this.
- covariate-constrained randomization is not reflected in the conventional analyses described nor are these conventional analyses justified. Preliminary methodological work may be required.
- how will repeated measures analyses be handled? What if trajectories are not "straight-line"? This needs additional thought (or at least additional specification as a supporting analysis).

**Review Type / Type d'évaluation:**	Reviewer 3 / Évaluateur 3
**Name of Applicant / Nom du chercheur:**	Miller, Jordan
**Application No. / Numéro de demande:**	419558
**Agency / Agence:**	CIHR/IRSC
**Competition / Concours:**	Project Grant/Subvention Projet
**Committee / Comité:**	Randomized Controlled Trials 2/Essais contrôlés randomisés 2
**Title / Titre:**	Determining the impact of a physiotherapist-led primary care model for low back pain - A cluster randomized controlled trial

Budget Recommendation/Recommandation budgétaire:

$1.35M over 4 years, 20 sites, 1560 pts.

Accepted, as described.

Please indicate your appraisal of the integration of sex as a biological variable as a strength, weakness, or not applicable to the proposal./Prière de sélectionner une option pour donner votre évaluation de l'intégration du sexe comme variable biologique en tant que point fort ou point faible de la proposition, ou en tant qu'élément non applicable à la proposition.

- (●) **Strength/Point fort**
- ( ) **Weakness/Point faible**
- ( ) **Not applicable/Non applicable**

Please indicate your appraisal of the integration of gender as a socio-cultural determinant of health as a strength, weakness, or not applicable to the proposal./Prière de sélectionner une option pour donner votre évaluation de l'intégration du genre comme déterminant socioculturel de la santé en tant que point fort ou point faible de la proposition, ou en tant qu'élément non applicable à la proposition.

- (●) **Strength/Point fort**
- ( ) **Weakness/Point faible**
- ( ) **Not applicable/Non applicable**

**Review Type / Type d'évaluation:**	Reviewer 3 / Évaluateur 3
**Name of Applicant / Nom du chercheur:**	Miller, Jordan
**Application No. / Numéro de demande:**	419558
**Agency / Agence:**	CIHR/IRSC
**Competition / Concours:**	Project Grant/Subvention Projet
**Committee / Comité:**	Randomized Controlled Trials 2/Essais contrôlés randomisés 2
**Title / Titre:**	Determining the impact of a physiotherapist-led primary care model for low back pain - A cluster randomized controlled trial

Sex and/or Gender Considerations/Notions de sexe et/ou de genre:

Sex and gender are both considered as part of randomization and design, as well as at the analysis and interpretation.

Impact of sex and gender on implications and translation of findings into practice are well explained.

Review Type/Type d'évaluation: SO Notes /Notes de l'agent scientifique
Name of Applicant/Nom du chercheur: Miller, Jordan
Application No./Numéro de demande: 419558
Agency/Agence: CIHR/IRSC
Competition/Concours: 2019-03-06 Project Grant/Subvention Projet
Committee/Comité: Randomized Controlled Trials 2/Essais contrôlés randomisés 2
Title/Titre: Determining the impact of a physiotherapist-led primary care model for low back pain - A cluster randomized controlled trial

---

Assessment/Évaluation:

Strengths:

This is a re-submission from an early career investigator. The proposal is novel and very well written. The overarching goal is clinically relevant and important. The proposal has the potential to decrease opioid prescriptions for LBP. The clustered randomized trial design method is well justified. Primary outcome has previously been well validated. The investigators have addressed all prior comments made by reviewers. The engagement of stakeholders including patients was felt to be an important strength. Gender and sex issues were particularly well justified and clear. Finally, investigators have also performed a pilot randomized trial (manuscript submitted) and feasibility is not a concern

Weaknesses:

It was highlighted that only 3 PTs will be developing the plan, and this might affect generalizability. The committee also wondered if some of the evaluations could be blinded. Finally, more details on the knowledge translation plan would have strengthen the application.

Budget:

No concerns.

Note: The final rating of the application, provided in the Notice of Decision (NOD), is the averaged rating of the peer review committee members following the discussion of the application during the committee meeting, and therefore may differ from the ratings provided by the assigned reviewers in their respective reviews.

``````````````````````````````````````````````````````````````````````````````````````````````````````````````````````````
``````````````````````````````````````````````````````````````````````````````````````````````````````````````````````````

**Review Type/Type d'évaluation:**	SO Notes /Notes de l'agent scientifique
**Name of Applicant/Nom du chercheur:**	Miller, Jordan
**Application No./Numéro de demande:**	419558
**Agency/Agence:**	CIHR/IRSC
**Competition/Concours:**	2019-03-06 Project Grant/Subvention Projet
**Committee/Comité:**	Randomized Controlled Trials 2/Essais contrôlés randomisés 2
**Title/Titre:**	Determining the impact of a physiotherapist-led primary care model for low back pain - A cluster randomized controlled trial

Assessment/Évaluation:

Remarque : La cote définitive de la demande, qui apparaît dans l'avis de décision, représente la moyenne des cotes accordées par les membres du comité d'évaluation par les pairs après avoir débattu de la demande à la réunion du comité. Elle peut donc différer de celle donnée par les évaluateurs dans leur évaluation respective.

Canadian Institutes of Health Research | Instituts de recherche en santé du Canada

AUTHORIZATION FOR FUNDING

CIHR (Canadian Institutes of Health Research) has approved funding as detailed below. Subject to the approbation of funding by Parliament, these funds will be made available to the business officer at the indicated institution for disbursement.

AUTORISATION DE FINANCEMENT

IRSC (Instituts de recherche en santé du Canada) vous accorde les fonds tel qu'indiqué ci-dessous. Suivant l'affectation des crédits par le Parlement du Canada, les fonds seront mis à la disposition du trésorier de l'établissement indiqué qui s'occupera des versements.

201903PJT-419558-RC2-CEDA-223355

17/07/2019

Institution Paid/Établissement chargé d'administrer les fonds:
Queen's University (Kingston, Ontario)

Recipient(s)/Bénéficiaire(s): Miller, Jordan
Rehabilitation Therapy
Faculty of Health Sciences
Queen's University (Kingston, Ontario)

Program/Programme: Project Grant
Grant New

Primary Institute/Institut principal: Health Services and Policy Research

Project Title/Titre du projet:

Determining the impact of a physiotherapist-led primary care model for low back pain - A cluster randomized controlled trial

Co-investigator(s) & Associates/Supervisor(s)/Host/Co-chercheur(s)/Directeur(s) de recherche/Hôte:

Dr. Julie Anne Richardson, Dr. Garry Salisbury, Dr. Monica Taljaard, Dr. Jacqueline Lee Whittaker, et al.

PAYMENT DETAILS/DÉTAILS DES VERSEMENTS — Funding Reference Number/ No. de Référence du financement: **PJT — 166177**

Period Période	Type	Amount by Type Montant par type	Total by Fiscal Year Total par exercice	
01/10/2019 to 31/03/2020	Operating	$175,472	$175,472	2019-20
01/04/2020 to 31/03/2021	Operating	$350,944	$350,944	2020-21
01/04/2021 to 31/03/2022	Operating	$350,944	$350,944	2021-22
01/04/2022 to 31/03/2023	Operating	$350,944	$350,944	2022-23
01/04/2023 to 30/09/2023	Operating	$175,472	$175,472	2023-24

Progress Report Required: Rapport des progrès réalisés requis: **Not Applicable**	Application to Renew Funding Required: Demande de renouvellement des fonds requis: **Non-Renewable**

NOTES:

CIHR requires that its contribution to your research be acknowledged in all written and oral presentations of your research results, including scientific articles, news releases, news conferences, public lectures and media interviews. For all scientific articles, the CIHR acknowledgement must include your Funding Reference Number (FRN) indicated above in the "Payment Details" section. Please see CIHR's Guidelines on Public Communication which are enclosed for more information on public communication and acknowledging requirements.

You received this funding because your colleagues volunteered their time to assist CIHR with the review of your application. We ask that, as a recipient of CIHR funding, you will participate in CIHR peer review activities if invited.

By drawing on the funds provided through this grant/award you agree to the terms and conditions set out in the attached "Conditions of Funding", any breach of which may result in CIHR taking remedial action as described therein.

If you are in receipt or become eligible to receive any funding from another source for any part of this project, you must advise CIHR immediately by following the instructions outlined in the "Funding Overlap Declaration" form http://www.cihr-irsc.gc.ca/e/797.html. Failure to self-declare overlap could lead to CIHR cancelling all funding related to this grant.

CIHR will require you to submit an electronic Final Report through the Research Reporting System on ResearchNet for this grant. Instructions will be provided through an email notification from the ResearchNet system once the activity becomes available.

Renée Venne

Renée Venne
Acting Manager, Contact Centre
Operations Support

cc
___ Supervisor/Directeur
___ Dean/Doyen
___ Host/Hôte
___ Administration
___ Accountant/Comptable
___ CIHR Finance/Service des Finance d'IRSC
___ Other/Autre

Canada

Queen's University Health Sciences & Affiliated Teaching Hospitals Research Ethics Board (HSREB)

HSREB Initial Ethics Clearance

March 04, 2020

Dr. Jordan Miller
School of Rehabilitation Therapy
Queen's University

TRAQ #: 6027847
Department Code: REH-755-19
Study Title: "REH-755-19 Determining the impact of a new physiotherapist-led primary care model for low back pain - a cluster randomized controlled trial"
Co-Investigators: Dr. Michael E Green, Dr. Simon French, Dr. Kathleen E Norman, Dr. David Barber, Dr. Catherine Donnelly, Mr. Kyle Vader
Review Type: Delegated
Date of Full Board Meeting: November 11, 2019
Date Ethics Clearance Issued: March 04, 2020
Ethics Clearance Expiry Date: March 04, 2021

Dear Dr. Miller:

The Queen's University Health Sciences & Affiliated Teaching Hospitals Research Ethics Board (HSREB) has reviewed the application and granted ethics clearance for the documents listed below. Ethics clearance is granted until the expiration date noted above.

Document Name	Comments	Version Date
Protocol	Revised Protocol - Clean	2019/12/19
Letter of Information/Consent Form (combined document)	FINAL LOI and Consent Form clean	2020/03/04
Questionnaire	Revised 12-Month Follow-Up Questionnaire with Tracked Changes	2019/12/19
Questionnaire	Revised 9-Month Follow-Up Questionnaire with Tracked Changes	2019/12/19
Questionnaire	Revised 6-Month Follow-Up Questionnaire with Tracked Changes	2019/12/19
Interview Guide	Interview Guide for Patients	2019/09/11
Interview Guide	Interview Guide for Health Care Providers	2019/09/11
Other document	Medical Record Abstraction Collection Form	2019/09/11

Documents Acknowledged:

Document Name	Comments	Version Date
Other document	Research Agreement with Clinical Sites	2019/12/19
Confidentiality Agreement	Data Sharing Agreement with Clinical Sites	2019/12/19
Other document	Scientific review - CIHR project grant reviews	2019/10/07

Document Name	Comments	Version Date
Other document	Budget	2019/09/11
Questionnaire	Keele STarT Back Tool	2019/09/11
Other document	Fidelity Checklist for Physiotherapy Assessments	2019/09/11
Ethics training certificates		2019/09/11
Principal Investigator CV	CV Jordan Miller	2019/09/11

Amendments: No deviation from, or changes to the protocol, informed consent form and conduct of study should be initiated without prior written clearance or an appropriate amendment from the HSREB, except when necessary to eliminate immediate hazard(s) to study participants or when the change(s) involves only administrative or logistical aspects of the study.

Renewals: An annual renewal event form or a study closure event form must be submitted annually as per the TCPS 2 Article 6.14. As a courtesy, the Office of Research Ethics may send reminders 30 days in advance of the ethics clearance expiry date. All lapses in ethics clearance will be documented on the annual renewal clearance letter. Suspension letters may be issued for lapses in ethics clearances one day or greater, with subsequent termination and closure of the ethics file for lapses greater than 10 business days. Terminations should be reported to applicable regulatory authorities (e.g., Health Canada, FDA).

Completion/Termination: The HSREB must be notified of the completion or termination of this study through the submission of a study closure event in TRAQ.

Reporting of Serious Adverse Events: Any unexpected serious adverse events occurring locally must be reported within 2 working days or earlier if required by the study sponsor. All other serious adverse events must be reported within 15 days after becoming aware of the information.

Reporting of Complaints: Any complaints made by participants or persons acting on behalf of participants must be reported to the Research Ethics Board within 7 days of becoming aware of the complaint.

Note: All documents supplied to participants must have the contact information for the Research Ethics Board.

Investigators please note that if your study is registered by the sponsor, you must take responsibility to ensure that the registration information is accurate and complete.

Regards,

Albert F. Clark.

Albert F. Clark, PhD
Chair, Queen's University Health Sciences and Affiliated Teaching Hospitals Ethics Board

The HSREB operates in compliance with, and is constituted in accordance with, the requirements of the Tri-Council Policy Statement: Ethical Conduct for Research Involving Humans (TCPS 2); the international Conference on Harmonisation Good Clinical Practice Consolidated Guideline (ICH GCP); Part C, Division 5 of the Food and Drug Regulations; Part 4 of the Natural Health Product Regulations; Part 3 of the Medical Devices Regulations, and the provisions of the Ontario Personal Health Information Protection Act (PHIPA 2004) and its applicable regulations. The HSREB is qualified through the CTO REB Qualification Program and is registered with the U.S. Department of Health and Human Services (DHHS) Office for Human Research Protection (OHRP). Federalwide Assurance Number: FWA#: 00004184, IRB#: 00001173. HSREB members involved in the research project do not participate in the review, discussion or decision.
